# A Gene Island with Two Possible Configurations Is Involved in Chromatic Acclimation in Marine *Synechococcus*

**DOI:** 10.1371/journal.pone.0084459

**Published:** 2013-12-31

**Authors:** Florian Humily, Frédéric Partensky, Christophe Six, Gregory K. Farrant, Morgane Ratin, Dominique Marie, Laurence Garczarek

**Affiliations:** 1 Université Pierre et Marie Curie (Paris VI), Station Biologique, Roscoff, France; 2 Centre National de la Recherche Scientifique (CNRS), UMR 7144, Oceanic Plankton group, Marine Phototrophic Prokaryotes team, Roscoff, France; Universidad Miguel Hernandez, Spain

## Abstract

*Synechococcus*, the second most abundant oxygenic phototroph in the marine environment, harbors the largest pigment diversity known within a single genus of cyanobacteria, allowing it to exploit a wide range of light niches. Some strains are capable of Type IV chromatic acclimation (CA4), a process by which cells can match the phycobilin content of their phycobilisomes to the ambient light quality. Here, we performed extensive genomic comparisons to explore the diversity of this process within the marine *Synechococcus* radiation. A specific gene island was identified in all CA4-performing strains, containing two genes (*fciA*/b) coding for possible transcriptional regulators and one gene coding for a phycobilin lyase. However, two distinct configurations of this cluster were observed, depending on the lineage. CA4-A islands contain the *mpeZ* gene, encoding a recently characterized phycoerythrobilin lyase-isomerase, and a third, small, possible regulator called *fciC*. In CA4-B islands, the lyase gene encodes an uncharacterized relative of MpeZ, called MpeW. While *mpeZ* is expressed more in blue light than green light, this is the reverse for *mpeW*, although only small phenotypic differences were found among chromatic acclimaters possessing either CA4 island type. This study provides novel insights into understanding both diversity and evolution of the CA4 process.

## Introduction

Light is one of the main factors affecting growth and photosynthetic rates of marine phytoplankton. In the field, this resource varies not only in quantity according to diurnal or seasonal cycles, but also in spectral quality both horizontally, along coast-offshore gradients, and vertically since the wavelengths of the solar spectrum are differentially filtered out throughout the water column, with blue light penetrating the deepest [1,2]. Competition between photosynthetic organisms for light has triggered the evolution of an amazing variety of pigments and chromophorylated proteins and different capabilities for sensing and responding to changing light conditions. Cyanobacteria evolved particularly sophisticated light-harvesting systems called phycobilisomes [3]. These are made of a complex combination of phycobiliproteins, which covalently bind chromophores, known as phycobilins, to cysteinyl residues (for reviews, see e.g. 4,5). Each phycobiliprotein α- and β-subunits may bind one to three phycobilins, which can be either phycocyanobilin (PCB), phycoerythrobilin (PEB) or phycourobilin (PUB; [6,7]). PCB efficiently captures orange photons (a_max_^~^ 620 nm), while PEB absorbs maximally in the green (a_max_^~^ 545 nm) and PUB in the blue (a_max_^~^ 495 nm). The latter pigment is considered to be the most abundant phycobilin in the ocean, due to the prevalence of blue photons in the euphotic zone [8,9]. Phycobiliproteins from oceanic cyanobacteria, including the coccoids *Synechococcus* and *Crocosphaera* and the filamentous *Trichodesmium*, are particularly rich in PUB, and this clearly represents an important adaptive event for life in offshore, blue waters [7,8,10].

Within the cyanobacterial community, *Synechococcus* is numerically the second most abundant oxygenic phototroph [11-13], contributing significantly to global primary productivity [14,15]. This genus harbors the largest pigment diversity of all marine cyanobacteria, explaining its ability to colonize a wide range of light niches, from turbid coastal waters to the most transparent waters of open ocean [9,16,17]. This diversity is due to the numerous possible combinations of phycobiliproteins with various phycobilins that constitute the phycobilisome rods [7]. While the simplest rods (pigment type 1) contain only phycocyanin, pigment type 2 strains also include one phycoerythrin form (PEI). However, most marine *Synechococcus* have two distinct forms of phycoerythrin, called PEI and PEII, and have been classified into pigment type 3, which is subdivided into four pigment subtypes according to the PUB:PEB ratio of whole cells [7]. The latter is generally assessed by the ratio of the relative fluorescence excitation maxima of these two phycobilins (Exc_495:545_), which is either low (~ 0.4), medium (~ 0.8) or high (> 1.7) in subtypes 3a, 3b or 3c, respectively [7]. The fourth subtype (3d) corresponds to the most sophisticated pigmentation found in marine *Synechococcus*, i.e. cells able to tune their pigmentation according to the ambient spectral light quality, a process termed CA4 (for Type IV chromatic acclimation; [18-20]). Most genes involved in the biosynthesis of phycobilisome rods are present in a large genomic cluster called the phycobilisome rod region, the gene content of which is specific to the pigment type [7], but prior to the present work it was not known whether genes involved in CA4 were also grouped into a cluster.

CA4 occurs during shifts from green (GL) to blue light (BL) or reciprocally [18,19]. Unlike other CA types (i.e. CA2 and CA3; [21-23]), CA4 does not seem to involve a significant alteration of the phycobiliprotein composition of phycobilisomes, but rather changes in chromophore content at three sites within PE [19,20]. In *Synechococcus* sp. RS9916, of the five chromophores bound to cysteinyl residues of PEI, only the one attached to Cys-α-139 is altered by this process, while it affects two of the six phycobilins bound to PEII, at Cys-α-83 and Cys-α-140. These sites are chromophorylated with PUB in BL and PEB in GL. Recently, the function of MpeZ was characterized in *Synechococcus* sp. RS9916 and identified as one of the key enzymes involved in the CA4 process [20]. MpeZ is bi-functional, since it can both attach a PEB chromophore to PEII α-83 and isomerize it to PUB. The *mpeZ* gene encoding this lyase-isomerase is expressed six-fold more in BL than GL and its inactivation strongly altered the CA4 response [20].

CA4 appears to be widespread within the marine *Synechococcus* radiation, since it has been reported in at least four distinct clades of subcluster 5.1, the most abundant and diversified *Synechococcus* group [7,18,24]. However, the true extent of this process within this lineage, as well as its genetic and phenotypic variability, have not been explored yet. Here, we did a comparative genomics study of a wide set of marine *Synechococcus* strains performing CA4 and identified a genomic island whose presence is correlated with the ability to carry out this process. A physiological characterization of strains that possess part of or the entire island was also performed, which allowed us to highlight some interesting phenotypic differences in the acclimation kinetics and/or amplitudes of the CA4 response among strains, and to refine the classification of marine *Synechococcus* spp. into different pigment types. 

## Results

### Discovery of a gene island correlated with CA4 exhibiting two different configurations

The availability of the whole genome sequence of 15 marine *Synechococcus* isolates and preliminary data from 25 additional *Synechococcus* unpublished genomes (that will be described in detail elsewhere; G. Farrant and coworkers, unpublished) allowed us to search for genes potentially involved in CA4. Such genes were retrieved in 16 of these 40 sequenced strains and one additional unsequenced strain (M11.1), originating from a variety of oceanic environments and depths (*cf*. Table S1). This set encompasses 15 strains belonging to six distinct clades within subcluster 5.1 (or ten subclades, as defined using the high resolution marker gene *petB* [24]; and two strains from a single clade of subcluster 5.3 (sensu [25]. In contrast, there were no strains from subcluster 5.2, since all those included in the initial dataset lack phycoerythrin and are thus incapable of CA4.

**Figure 1:**
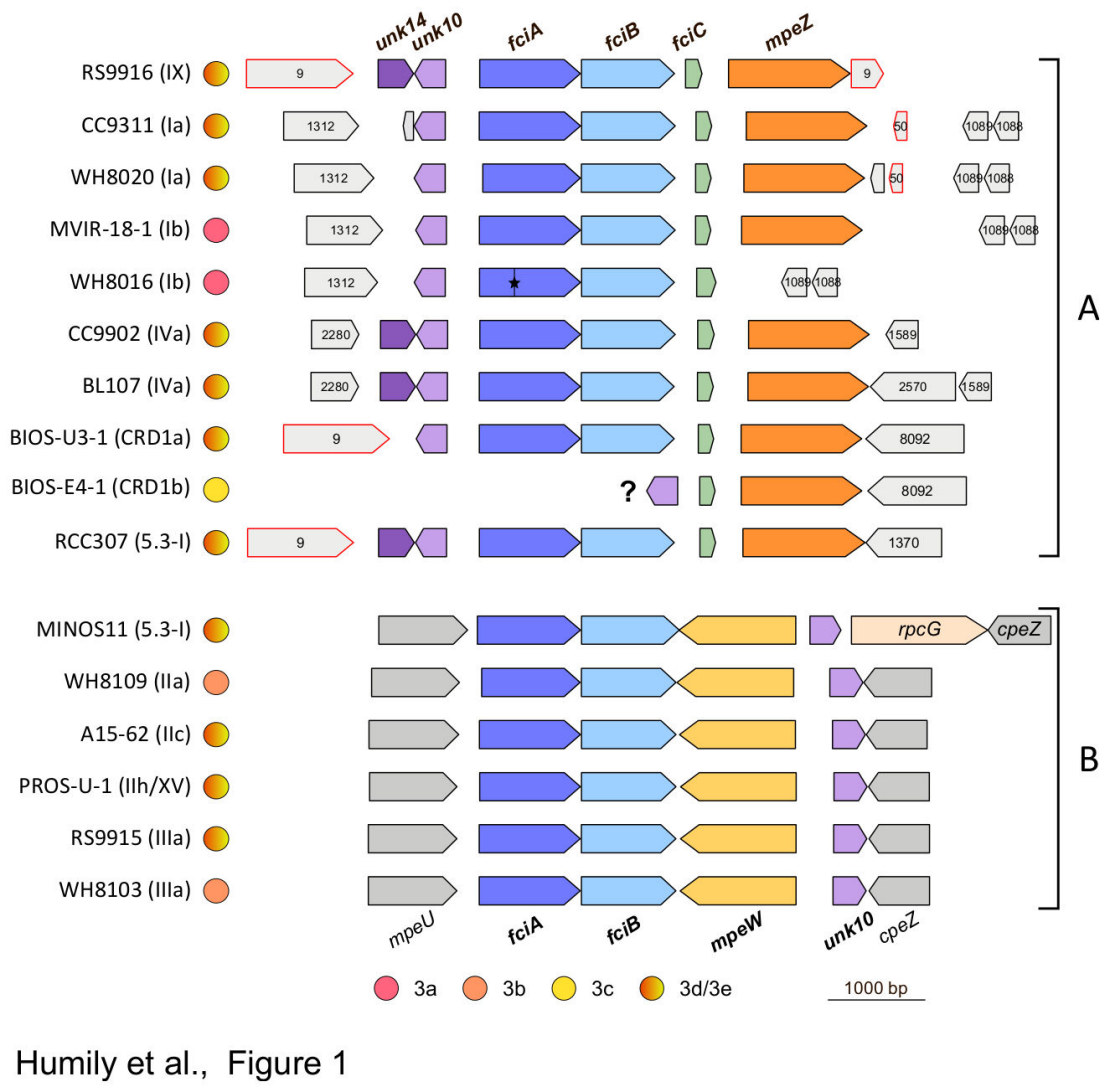
Gene content and genomic context of the two types of CA4 gene islands found in marine *Synechococcus* genomes. The CA4-A island can be found at different locations of the genome (though the context is the same within a given clade), whereas the CA4-B island is always located in the main phycobilisome region between *mpeU* and *cpeZ* genes. The gene size and organization are represented to scale (scale bar = 1000 bp) and all regions aligned with regard to the *fciA* gene (except for BIOS-E4-1, aligned on *mpeZ* of BIOS-U3-1). Genes included in CA4 regions are shown in color and orthologs are indicated with the same color. Short CA4 genes of unknown function are named according to [7], with *unk14* and *fciC* being novel genes, specific of the CA4-A region. Genes found in the immediate vicinity of CA4 regions (shown in grey) are designated either by their gene names or their cluster number in the Cyanorak v2.0 database of orthologous sequences (http://www.sb-roscoff.fr/Phyto/cyanorak/; with the prefix CK_ being omitted). One stop codon interrupting WH8016 *fciA* is indicated by a star. The phylogenetic affiliation of strains, as reported in previous studies (see text), is mentioned between brackets. The pigment type is indicated by circles bi-colored for strains that chromatically adapt and uni-colored for those that do not (see Table 1 for details). Hotspots for DNA recombination, *hli* (Cluster CK_00000050) and *psbA* (Cluster CK_00000009), are bordered by a red line.

The 16 sequenced strains containing genes putatively involved in CA4 were identified using a two-step bioinformatic approach. First, the 40 genomes were screened for the presence of *mpeZ* (Cyanorak cluster CK_00009110; see www.sb-roscoff.fr/cyanorak/), a gene that encodes a phycobilin lyase-isomerase recently shown to have a key role in CA4 in *Synechococcus* RS9916 [20]. Orthologs of this gene were retrieved in 8 additional genomes, where the *mpeZ* gene was consistently located at the 3'-end of a genomic island (hereafter called "CA4-A island"), always downstream of three additional in-frame genes (Figure 1). Structural analysis using Phyre2 [26] predicted that the first two genes encode proteins (Cyanorak clusters CK_00002124 and CK_00002123), which both possess a possible C-terminal α-helix-turn-α-helix (HTH) DNA binding domain (IPR018060; Figures S1A and B). This domain, known to consist of seven alpha helices, is also found in a large family of proteins whose founding member is AraC, a transcriptional activator involved in the control of the arabinose metabolism in *Escherichia coli* [27]. The third gene of the CA4-A island (Cyanorak cluster CK_00009137) is predicted to encode a protein of 55-67 aa and characterized by a N-terminus with a predicted conserved ribbon-helix-helix domain (IPR010985), also found in several bacterial or phage repressors (Figure S1C; [28]). Thus, together with the two *araC*-like gene products, the protein encoded by this gene may be involved in regulating the CA4 process. Based on their genomic context and position just upstream of and in the same reading frame as the CA4-specific phycobilin lyase *mpeZ* [20], we propose to call these three genes *fciA-C* (where "*fci*" stands for "type four chromatic acclimation island"; Figure 1). Another gene of unknown function was consistently found at the 5'-end of the CA4 island, but was encoded on the reverse strand relative to the *fci* genes. This gene is an ortholog of *unk10* (Cyanorak cluster CK_00002279), previously observed in the phycobilisome rod gene region of pigment type 3c (i.e. high-PUB) strains WH8102 and CC9605, specifically at the 5'-end of the PEII sub-region (see Figure 6 in [7]). Unk10, predicted to be 107-118 aa in length, contains a ^~^43 aa N-terminal Nif11 domain (IPR012903; e-value: 2.3 10^-8^), so-called because it was initially found in the product of an uncharacterized gene of the *nif* cluster of *Azotobacter vinelandii* [29]. In four out of the nine *mpeZ*-containing strains (RS9916, BL107, CC9902 and RCC307), another gene, encoding a predicted 121-123 aa protein of unknown function, was present upstream and head-to-head relative to *unk10* (Figure 1). This gene, which was designated *unk14* (to extend the numbering of unknown genes in the phycobilisome rod region proposed by [7]) (Cyanorak cluster CK_00009135), is specific to these four strains, but the encoded protein is paralogous to a protein of unknown function, whose gene is present in all marine *Synechococcus* strains (Cyanorak cluster CK_00000072). Unk14 belongs to the highly conserved protein family DUF2237 (or PF09996), which is widely distributed among Bacteria.

As a second step, we searched for the *fciA* and *fciB* genes in other *Synechococcus* genomes. This led to the discovery of one additional CA4-A strain lacking *mpeZ* (WH8016) as well as another type of CA4 island in six additional genomes (Figure 1). In contrast to the first one, this so-called "CA4-B island" lacks both *fciC* and *mpeZ*. The latter gene is absent, and in this location but on the opposite strand of *fciAB*, is a gene encoding a novel member of the phycobilin lyase E/F family [30,31]. Like MpeZ, it is a paralog of MpeY [32], whose gene is found in all PEII-containing *Synechococcus* strains and located in the PEII subregion of the phycobilisome rod region (Figure S2; see also Figure 6 in [7]). Since this gene is not a direct ortholog of *mpeZ* (see phylogenetic analysis below) and co-occurs with *mpeY*, we propose to call it *mpeW*. 

Other noticeable differences between CA4-A and CA4-B islands include the different locations of *unk10* with regard to the *fciAB* genes (upstream or downstream, respectively) and their distinct genomic context (Figure 1). Indeed, whatever the clade, the genomic context of the CA4-B island was always the same, i.e. in the middle of the phycobilisome rod gene region and more specifically between *mpeU* and *cpeZ*, at the 3'-end of the PEII sub-region (Figure S2). In contrast, even though the location of the CA4-A island was globally conserved among strains of a given clade, it differed between clades and, in all strains examined so far, was never located in the phycobilisome region (see Figures 1 and S1). Another important point is that within subcluster 5.1, all strains of a given clade possessed one type of CA4 island but not the other. Indeed, strains belonging to clades I, IV, IX and CRD1 possess a CA4-A island, while a CA4-B island was found in clades II and III. Although the genome of the chromatically acclimating strain *Synechococcus* sp. strain M11.1 is not available yet, we used PCR amplification and determined that it contains *mpeW* and not *mpeZ* (Figure S3), as expected for a clade II strain. Interestingly, this clade differentiation does not hold true for subcluster 5.3, since the closely related strains RCC307 and MINOS11 possess a CA4-A and a CA4-B island, respectively. 

### Phylogenetic analyses of genes putatively involved in CA4

**Figure 2:**
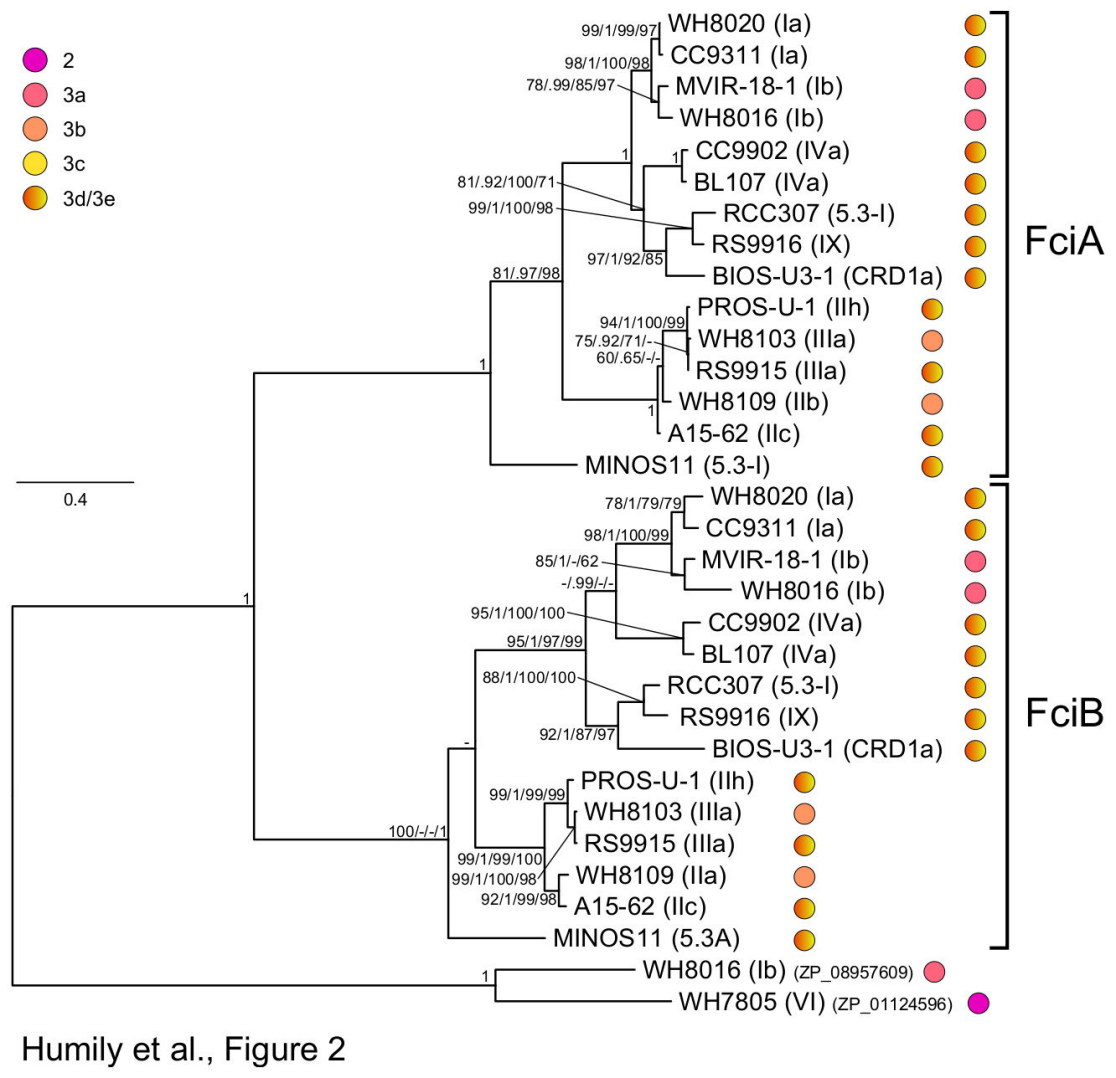
Maximum likelihood analysis of AraC-like proteins (289 aa positions) from marine *Synechococcus*. For each strain, the phylogenetic affiliation is mentioned between brackets and the pigment type is indicated by colored circles (see Table 1 for details). Series of four numbers shown at nodes correspond to bootstrap values for ML analyses, Bayesian posterior probabilities (PP, ranging between 0 and 1), and bootstrap values for Neighbor-Joining and Parsimony methods, respectively. Only values higher than 0.60 for PP and 60% for bootstrap values are shown on the phylogenetic tree. The scale bar represents 0.4 substitutions per amino acid.

In order to better understand the evolution of the CA4 process and notably the origin of the differentiation into two distinct CA4 islands, we performed a number of phylogenetic analyses using genes that are common to the two island types CA4-A and CA4-B (*fciA, fciB*, *unk10* and *mpeW* or *mpeZ*). Figure 2 shows a phylogenetic tree based on FciA and FciB sequences and rooted with two more distant, uncharacterized members of the AraC family retrieved from marine *Synechococcus* spp. (Syn8016DRAFT_2956 and WH7805_05326; Cyanorak cluster CK_00002282). As for other cyanobacterial members of the AraC family, their homology with FciA and FciB mostly resides at the C-terminal HTH domain level (data not shown). Orthologs of both Fci proteins separated into two main branches, the first one including all strains that have a CA4-A island (except BIOS-E4-1, which lacks *fciAB* genes) and the second one encompassing five out of the six strains having a CA4-B island (Figures 1 and 2). The sixth strain is the subcluster 5.3 strain MINOS11 whose FciA and FciB are split apart from other sequences, consistently with the wide phylogenetic distance between members of subclusters 5.1 and 5.3 [25]. By contrast, the fact that both Fci sequences from the other subcluster 5.3 strain, RCC307, fall within subcluster 5.1 close to clade IX strain RS9916 strongly suggests that RCC307 might have acquired these genes by lateral transfer from a cell belonging to clade IX or a related lineage. 

A globally similar tree topology was retrieved for Unk10 (Figure S4), except that i) BIOS-E4-1 was present and clustered with BIOS-U3-1, another member of the CRD1 clade [24,33] and ii) MINOS11 was clearly located at the base of the branch encompassing all strains that also possess *mpeW* (i.e. CA4-B strains). Interestingly, the latter branch also included the pigment type 3c (i.e. high-PUB) strains WH8102 and CC9605, in which the *unk10* gene is located at the 3'-end of the PEII gene subregion [7], as in most CA4-B strains (Figure 1).

**Figure 3:**
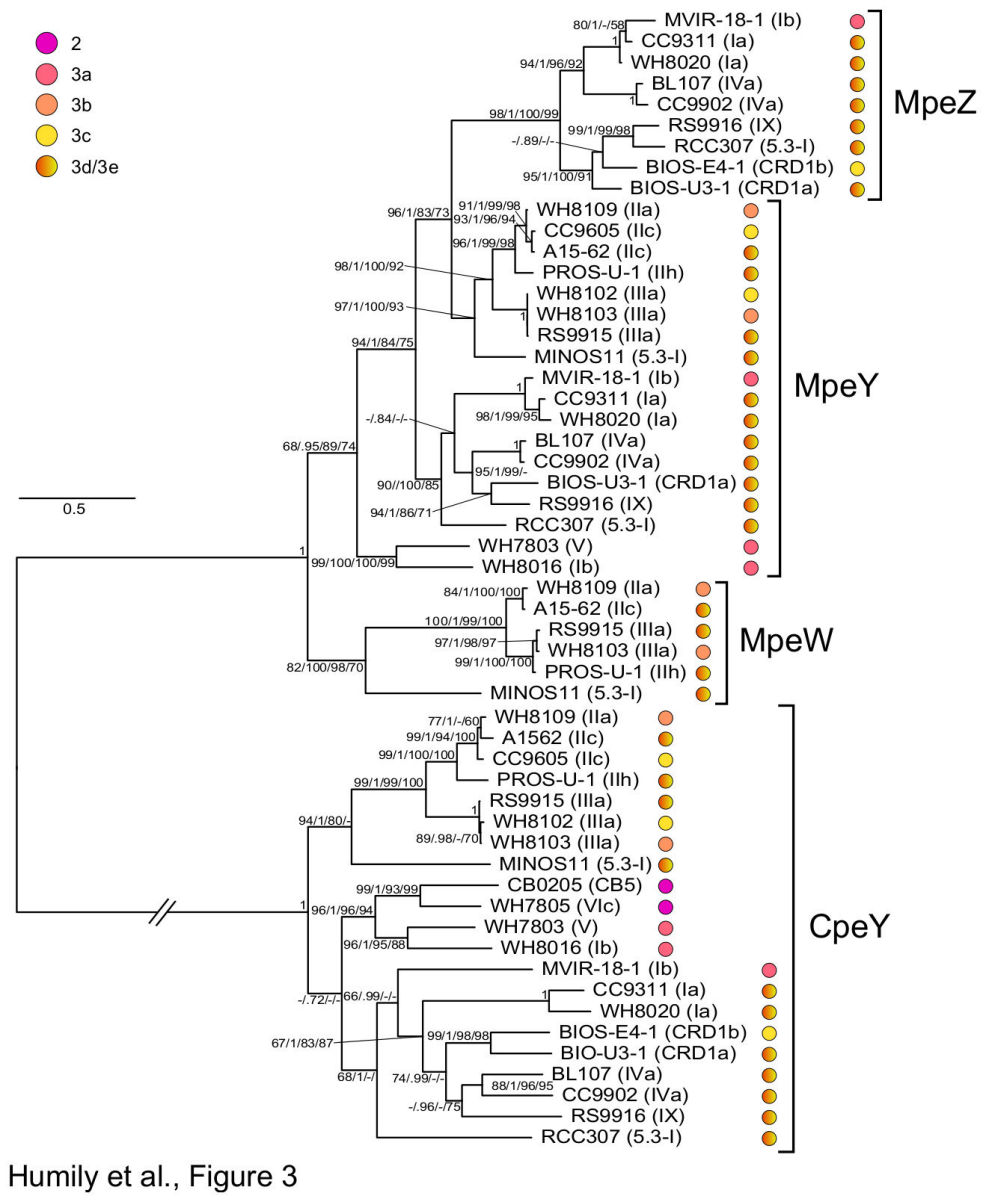
Maximum likelihood analysis of four phycobilin lyase sequences of the E/F clan (380 aa positions) from marine *Synechococcus*. For each strain, the phylogenetic affiliation is mentioned between brackets and the pigment type is indicated by colored circles (see Table 1 for details). Series of four numbers shown at nodes correspond to bootstrap values for ML analyses, Bayesian posterior probabilities (PP, ranging between 0 and 1), and bootstrap values for Neighbor-Joining and Parsimony methods, respectively. Only values higher than 0.60 for PP and 60% for bootstrap values are shown on the phylogenetic tree. The scale bar represents 0.6 substitutions per amino acid. The branch bearing the CpeY cluster was shortened for readability (its full length initially was 3.85 substitutions per amino acid).

We also generated an amino acid alignment comprising the sequences of MpeZ, MpeW and two other related members of the phycobilin lyases E/F clan [30], CpeY, occurring in all *Synechococcus* strains possessing PEI, and MpeY, occurring only in strains possessing PEII. While CpeY was biochemically characterized to catalyze the attachment of a PEB molecule at Cys-82 of the alpha-PEI subunit [34], MpeY is still uncharacterized [7,30]. Comparative motif analyses using Protomata (Figure S5; [35]) show that MpeW, MpeY and MpeZ share several conserved E-Z HEAT-repeat domains, known to facilitate protein-protein interactions [36,37], but also a few specific motifs, such as the strongly conserved 'HRDE', located around position 285 and not found in CpeY. In the phylogenetic tree shown in Figure 3, sequences of these three Mpe proteins grouped together, well apart from the CpeY branch. As for the Unk10 tree, MpeY sequences grouped into two main clusters, a first one containing all strains that possess *mpeZ* (i.e. a CA4-A island) and a second one with *mpeW*-containing and pigment type 3c strains. A third MpeY branch was also present and contained the pigment type 3a (i.e. low-PUB) strains WH7803 and WH8016. The occurrence of the latter strain at this position in the tree may seem surprising given that it possesses a partial (*mpeZ*-lacking) CA4-A island (Figure 1), but it is worth noting that its *mpeY* gene is located in a phycobilisome region whose gene content and organization is similar to that of WH7803 (Figure S2; see also Figure 6 in [7]).

A similar tree topology as for MpeY was found for CpeY (Figure 3), except that the third branch contained two pigment type 2 (i.e. PUB and PEII-less) strains, CB0205 and WH7805, which lack the *mpeY* gene. 

### Physiological characterization of *mpeW- or mpeZ*-containing strains acclimated to blue or green light

**Figure 4:**
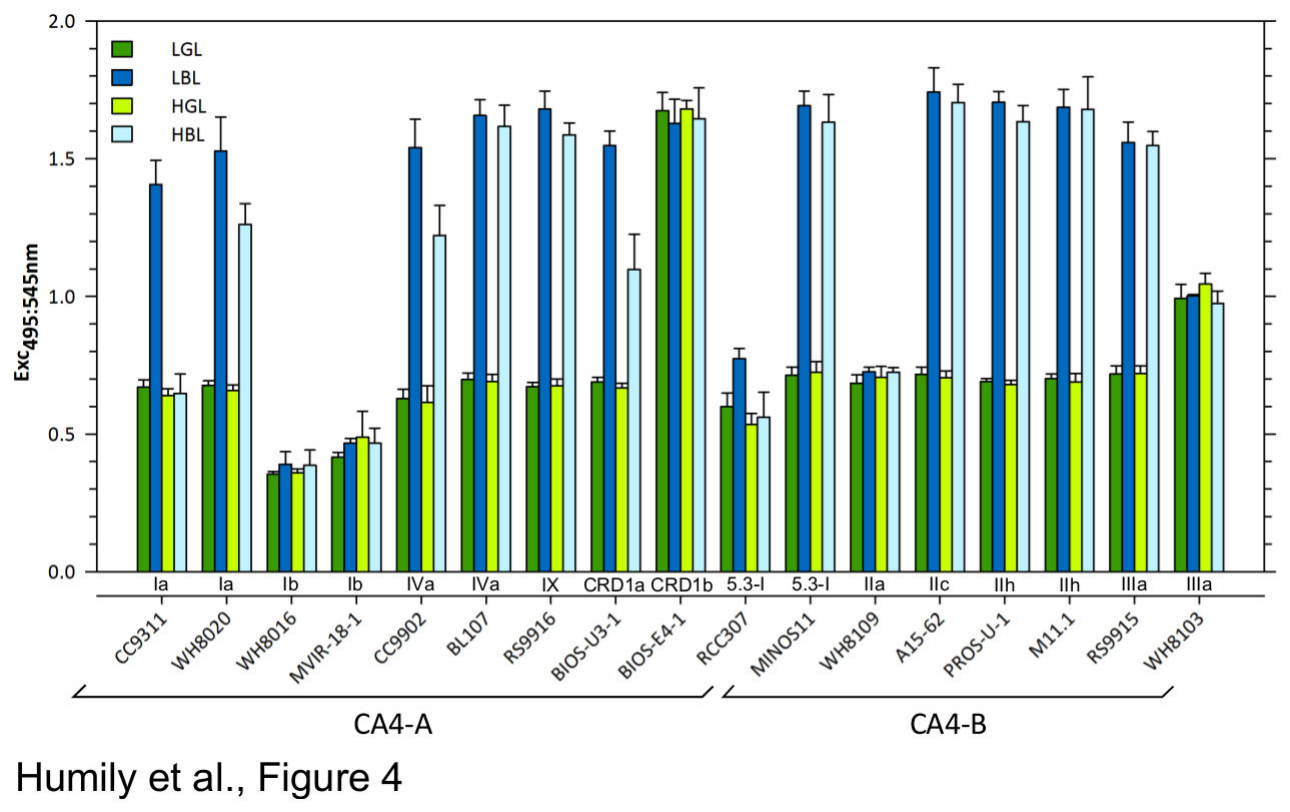
Average Exc_495:545_ ratios of CA4 genes-containing *Synechococcus* strains acclimated to blue light (BL) and green light (GL) at two different irradiances (20 and 75 µmol photons m^-2^ s^-1^). Low and high light conditions are shown respectively by dark and light colors (blue or green bars indicating BL and GL, respectively). A minimum of 5 replicates were used to calculate the mean and standard deviation.

In order to study the CA4 response of the different strains listed in Table S1, we measured the fluorescence Exc_495:545_ ratio, a proxy for the molar PUB to PEB ratio, from exponentially growing cultures acclimated to continuous blue (BL) or green (GL) light for over 20 generations (Figure 4). Measurements were done both on cultures grown at low light (LL; 20 µmol photons m^-2^ s^-1^) and high light (HL; 75 µmol photons m^-2^ s^-1^). The Exc_495:545_ ratio was generally stable in GL and more variable in BL (Figure 4). This observation held particularly true at high irradiance, at which the Exc_495:545_ ratio of BL controls often declined notably after cultures had reached the early stationary phase.

Despite the presence of genes potentially involved in CA4 in their genomes (see above), five strains (WH8103, BIOS-E4-1, MVIR-18-1, WH8016 and WH8109) were unable to chromatically acclimate, since they displayed a similar Exc_495:545_ ratio in GL and BL at both irradiances tested (Table 1). An additional strain (RCC307) seemingly had a strongly altered capacity for CA4, since it exhibited only a small increase (29%) of this ratio between low green (LGL) and blue (LBL) light (significant at Ρ<0.01; Figure 4). In contrast, for the latter strain, the Exc_495:545_ ratio was not significantly different between high green (HGL) and high blue (HBL) light (Ρ>0.1). However, the mean value of the Exc_495:545_ ratio (both irradiances considered, Table 1) was highly variable among the five non-chromatically acclimating strains since it was i) as low as measured for the pigment type 3a strain WH7803 [7] in WH8016 and MVIR-18-1 (0.37±0.04; n=30 and 0.46±0.06; n=27, respectively), ii) at a level intermediate between pigment type 3a and GL-grown CA4 strains for RCC307 (0.62±0.11, n=115), iii) similar to that of GL-grown CA4 strains for WH8109 (0.71±0.03, n=109), iv) intermediate between values measured in GL- and BL-grown CA4 strains for WH8103 (1.00±0.05, n=27) and v) comparable to that of BL-grown CA4 strains in BIOS-E4-1 (1.66±0.08, n=27).

**Table 1:** Summary of the genotypic and phenotypic pigment characteristics of the *Synechococcus* strains used in this study.

**Strains**	**Subclade^a^**	**Pigment type^b^**	**Pigment phenotype (this study)**	**CA4 island type**	**CA4 phenotypic group**	**Exc_495:545_ amplitude variations**	**Pigment type (this study)**
WH8016	Ib	3a	3a	A	na	None (ratio ~ 0.4)	3aA
MVIR-18-1	Ib	3a	3a	A	na	None (ratio ~ 0.5)	3aA
WH8103	IIIa	3c	3b	B	na	None (ratio ~ 1.0)	3bB
WH8109	IIa	3b	3b	B	na	None (ratio ~ 0.7)	3bB
BIOS-E4-1	CRD1b	3c	3c	B	na	None (ratio ~ 1.7)	3cB
RS9916	IX	3d	3d	A	2	Small variation in HBL	3dA
CC9311	Ia	3d	3d	A	2	Small variation in HBL	3dA
WH8020	Ia	3d	3d	A	2	Small variation in HBL	3dA
BIOS-U3-1	CRD1a	3d	3d	A	2	Small variation in HBL	3dA
CC9902	IVa	3d	3d	A	3	Large variation but delayed in BL to GL	3dA
BL107	IVa	3d	3d	A	3	Large variation but delayed in BL to GL	3dA
MINOS11	5.3-I	3d	3d	B	1	Large variation	3dB
A15-62	IIc	3d	3d	B	1	Large variation	3dB
PROS-U-1	IIh	3d	3d	B	1	Large variation	3dB
RS9915	IIIa	3d	3d	B	1	Large variation	3dB
M11.1	IIh	3d	3d	B?	1	Large variation	3dB?
RCC307	5.3-I	3b	3e	A	4	Small variation	3eA

Abbreviations: BL, blue light; GL, green light; HBL, high blue light; na, not applicable.

^a^ Phylogenetic affiliation following the nomenclature reported in previous studies [24,25,33,45,73]

^b^ Pigment type classification according to [7]

All eleven other strains analyzed in this study were able to match their pigmentation to the ambient light color with an average 134±13% increase of their Exc_495:545_ ratio between LGL and LBL, consistently with previous reports on CA4 [18-20]. A similar variation was also observed in HL (119±43%), except for CC9311, which maintained a low Exc_495:545_ ratio in HBL (Figure 4). It is noteworthy that the average Exc_495:545_ ratios were more variable and generally lower (*P*<0.01) for CA4-A strains than for their CA4-B counterparts in all four light conditions tested, though the difference was much more conspicuous in HL than LL (0.67±0.02 vs. 0.71±0.01 at LGL; 1.58±0.10 vs. 1.67±0.08 at LBL; 0.66±0.03 vs. 0.71±0.02 at HGL and 1.41±0.25 (excluding CC9311) vs. 1.63±0.06 at HBL, respectively). 

### CA4 induces a change in the expression of phycobilin lyase genes

**Figure 5:**
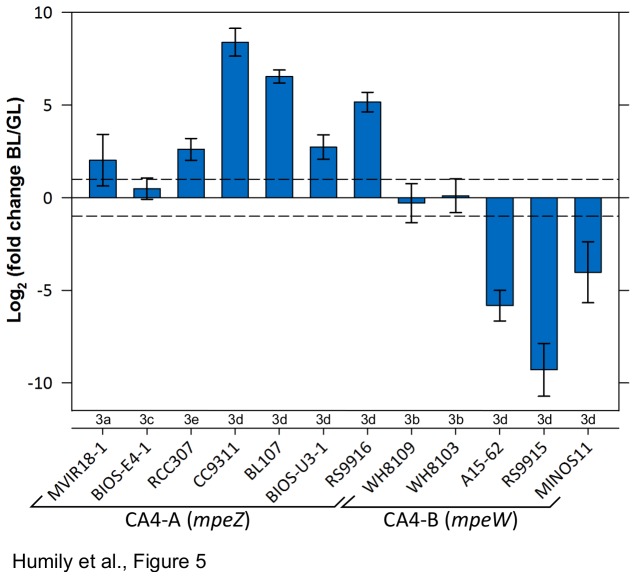
Differential expression of *mpeW* or *mpeZ* genes in *Synechococcus* cultures acclimated to BL *vs*. **GL as measured by real time PCR (20 µmol photons m^-2^ s^-1^ in both conditions)**. Mean and standard deviation were calculated from 3 biological replicates. Only differential transcript levels above or below the dotted lines (log_2_(FC) > 1 or <-1) were considered as significant.

The inability of *Synechococcus* spp. WH8016 and BIOS-E4-1 to acclimate to changes in light quality may be due to the absence of key CA4 genes in these genomes (i.e., *mpeZ* and *fciAB*, respectively; Figure 1). Less predictably, strains WH8109, WH8103, MVIR-18-1 and RCC307, which possess a complete set of CA4 genes (Figure 1), are also affected in their ability to perform CA4 (see above). Comparative analyses of protein sequences did not reveal any obvious mutation/deletion or insertion in the most conserved regions of FciA/B (data not shown) or MpeW/Z (Figure S4) that could explain this loss of CA4 phenotype. To check whether the absence of response could result from an alteration of the regulation of the transcript abundance of the characterized and putative CA4-specific lyase-encoding genes, *mpeZ* or *mpeW* transcript levels were measured in exponentially growing strains acclimated to either LGL or LBL in a representative selection of strains. Among the seven tested CA4-A strains, all four that exhibited a large variation of their Exc_495:545_ ratio between LGL and LBL (Figure 4) showed a significantly higher *mpeZ* expression in LBL than in LGL, ranging from 6.7-fold [log_2_(FC)=2.73±0.66] in BIOS-U3-1 up to 333-fold [log_2_(FC)=8.38±0.75] in CC9311 (Figure 5). RCC307, which showed only a small variation of its Exc_495:545_ ratio between LGL and LBL (Figure 4), also displayed a slightly higher expression of *mpeZ* in LBL than LGL, comparable to that of BIOS-U3-1 (Figure 5). In contrast, all three tested CA4-B strains that were capable of CA4 exhibited a significantly higher *mpeW* expression in LGL than in LBL, ranging from 35.9-fold [log_2_(FC)=4.03±1.65; n=3] in MINOS11 up to 1,150-fold [log_2_(FC)=9.29±1.43; n=3] in RS9915 (Figure 5). All strains that exhibited no significant change in their Exc_495:545_ ratio between BL and GL (Figure 4) also showed no significant difference in transcript abundances (t-test; *P*>0.05) for either the *mpeZ* or *mpeW* gene between these conditions, except for *Synechococcus* sp. MVIR-18-1, for which in one out of the three biological replicates *mpeW* was slightly more expressed in BL than GL. 

### Variability of growth and acclimation kinetics of CA4 strains during light quality shifts

**Figure 6:**
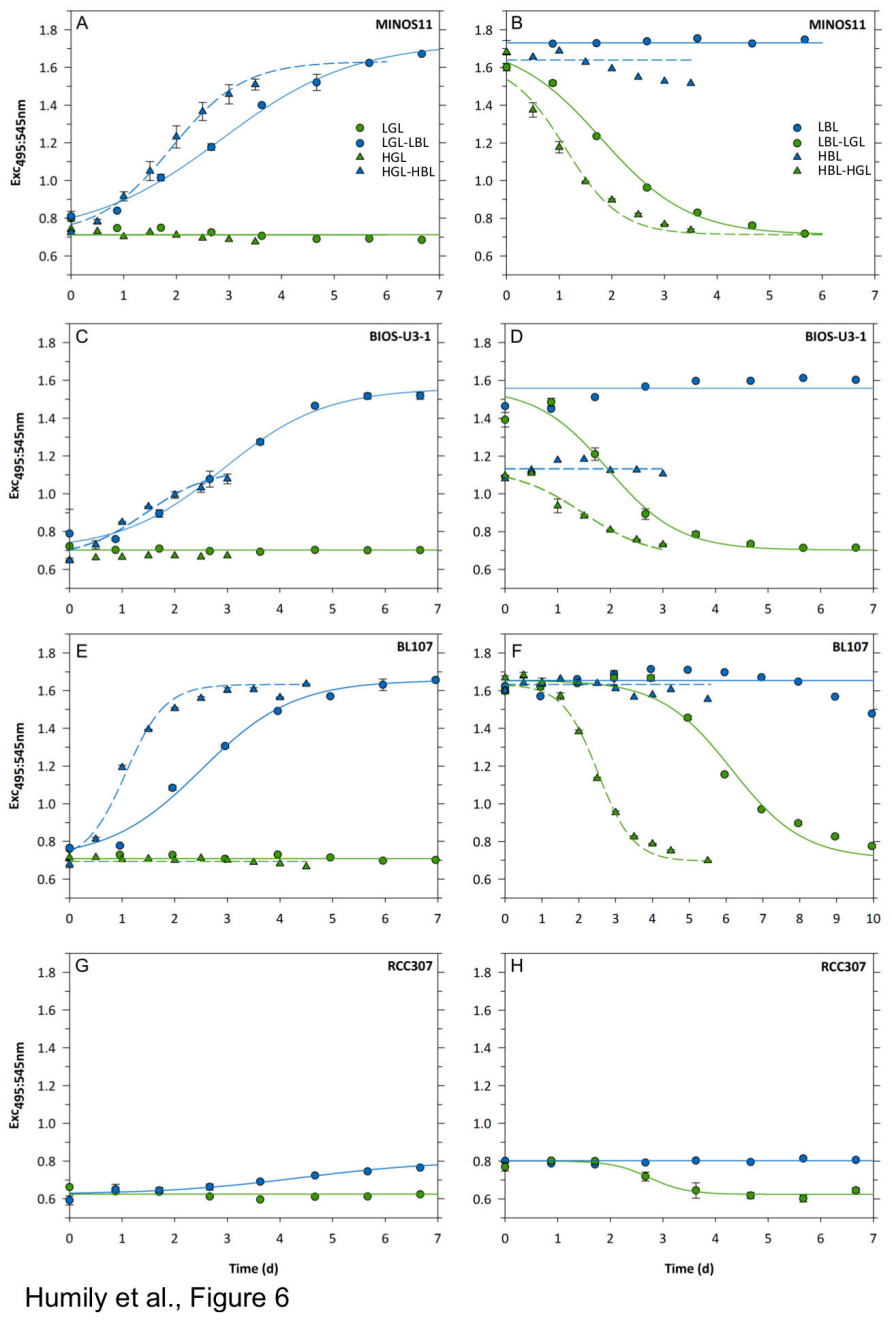
Phenotypic variability of chromatic acclimation in marine *Synechococcus*. Temporal changes of the Exc_495:545_ after shifts from BL to GL (and vice versa) at two irradiances: LL (circles; 20 µmol photons m^-2^ s^-1^) and HL (triangles; 75 µmol photons m^-2^ s^-1^). The color (blue or green) of lines and symbols matches the ambient light color under which cultures were shifted at time zero. Error bars indicate standard deviation for two biological replicates. Four distinct CA4 phenotypic groups were observed. (**A**,**B**) Group 1 strains: MINOS11, A15-62, PROS-U-1, M11.1 and RS9915; (**C**,**D**) group 2: BIOS-U3-1, CC9311, WH8020, CC9902 and RS9916; (**E**,**F**) group 3: BL107 and CC9902; (**G**,**H**): group 4 : RCC307. Note the different x-axis scale for BL107.

**Figure 7:**
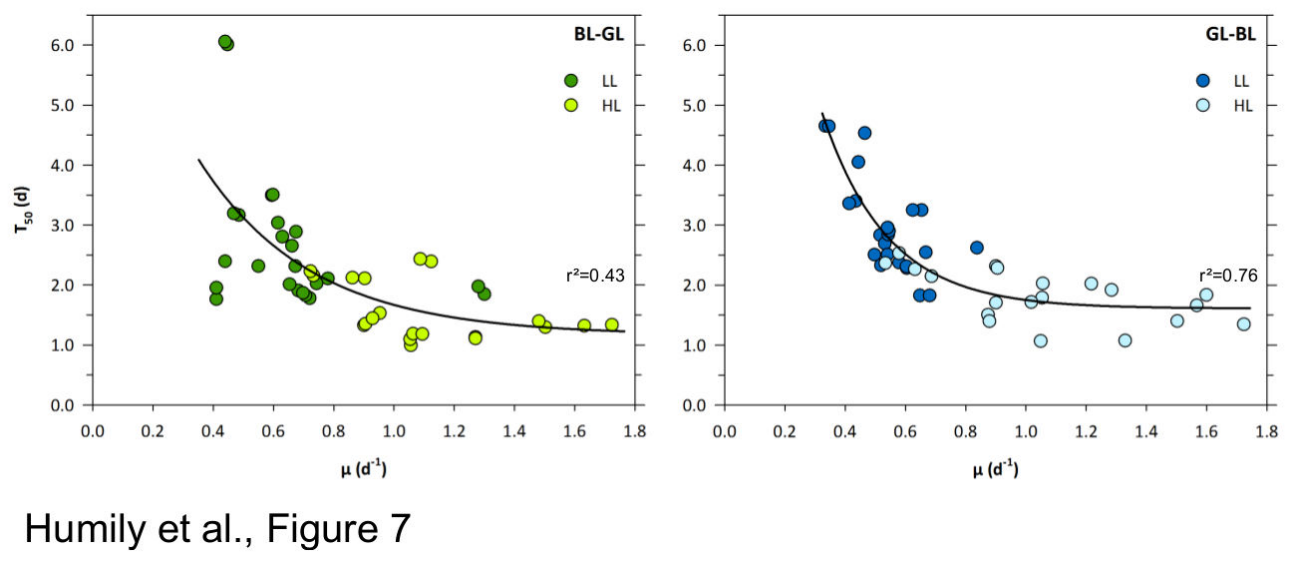
Correlations between growth rate (µ, days) and half maximal acclimation time (T_50_, days) for twelve *Synechococcus* strains. Shifts to another light quality (BL to GL and vice versa) were performed under two light intensities. LL and HL correspond respectively to 20 and 75 µmol photons m^-2^ s^-1^ of BL or GL. Data correspond to two biological replicates for each strain and each light condition.

The kinetics and/or amplitudes of chromatic acclimation of twelve strains, representative of six distinct clades of subcluster 5.1 and one clade of subcluster 5.3, were analyzed in order to reveal possible phenotypic differences. All strains were allowed to acclimate for at least one month in either BL or GL and at two irradiances, LL and HL, prior to being shifted to the other light color condition (same irradiance). Growth rates (µ) and the variation of the Exc_495:545_ ratio were then assessed from flow cytometric cell counts and by spectrofluorimetry, respectively. The latter measurements allowed us to assess the half acclimation time (T_50_ in days) for each strain and each type of light quality shift at both irradiances tested (see Table S2 and Figures 6 and 7). 

All strains displayed a significantly higher growth rate and a shorter T_50_ when grown in HL than in LL (Figure 7 and Table S2). Based on kinetics studies, four CA4 phenotypic groups can be defined among strains that showed a variable Exc_495:545_ ratio between GL and BL and Figure 6 illustrates one representative example (or the sole case) of each group. Group 1, comprising strains A15-62, PROS-U-1, M11.1, RS9915, and MINOS11, exhibited 'typical' CA4 kinetics (cf. 18-20), at both growth irradiances (Figure 6A-B and Table 1). This group exhibited i) significant changes in the Exc_495:545_ ratio within 1-2 days after the light color shift, ii) T_50_ values of 2.01±0.24 d for the LBL to LGL shift, 2.72±0.37 d for the reverse shift and 1.22±0.13 d and 1.85±0.36 d for the corresponding shifts at HL and iii) the complete acclimation to the new color took about 6 d at LL and this time was reduced to less than 4 d at HL (Figure 6). 

The second group, comprising the CA4-A strains BIOS-U3-1, CC9311, WH8020 and RS9916 was defined by the fact that cultures reached a significantly lower Exc_495:545_ at HBL than LBL, though their T_50_ was not significantly different from that of the previous group (*P*>0.05, Figure 6C-D and Table S2).

The third group comprises the two closely related strains BL107 and CC9902, which exhibited a systematic lag compared to the first two groups in the initiation of the CA4 process after being shifted from BL to GL. Indeed, the Exc_495:545_ ratio decreased only after 3-4 days in LL and 1.5-2 days in HL (Figure 6F). To better understand this behavior, we tested whether the delay in the CA4 initiation observed during the BL to GL shift was dependent on the acclimation time to BL before the shift to GL. We found that the delay was the same for BL107 cultures acclimated to BL for weeks as for cultures shifted to GL either immediately, or after 1 or 3 days after they reached the BL value of the Exc495:550 ratio following a GL to BL shift (Figure S6A and Table 1). Furthermore, BL107 cultures that were subjected to successive shifts, i.e. first from BL to GL, then back to BL just before or after being fully GL-acclimated, then again shifted from BL to GL immediately upon reaching the BL value of the Exc_495:545_ ratio, also exhibited the same delay (Figure S6B). Altogether, these experiments clearly demonstrate that this delay is independent of the light history of the culture. In contrast, no significant lag occurred after a shift of BL107 and CC9902 cultures from GL to BL (Figure 6E).

The fourth CA4 group comprises the sole strain RCC307, for which the amplitude of variation of the Exc_495:545_ ratio was very limited (0.63-0.80 for a shift from GL to BL; Figure 6G-H and Table 1). It is interesting to note that the Exc_495:545_ ratio in GL of this strain was slightly lower than that of other strains analyzed in this study. 

## Discussion

### Type 4 chromatic acclimation in marine Synechococcus requires a specialized genomic island

CA4 is a sophisticated light-controlled process by which *Synechococcus* spp. cyanobacteria can efficiently modify the phycobilin content of their phycobilisomes in response to fluctuations in light quality occurring in the marine environment along nutrient and/or light gradients [18-20]. In the present study, we have identified a small gene cluster comprising 4 to 6 genes, which appears to be indispensable and is correlated with the capacity of cells to carry out CA4. Unexpectedly, depending on *Synechococcus* strains, this cluster could exhibit two distinct configurations (so-called CA4-A and CA4-B) in terms of gene content, gene order and genomic context (Figure 1 and Table 1). Physiologically, chromatically acclimating strains containing either island type displayed a similar kinetics of variation of the Exc_495:545_ ratio in response to light color shifts, suggesting that CA4-A and CA4-B regions are functionally equivalent. However, while all CA4-B strains displayed a similar amplitude of variation of their Exc_495:545_ ratio at LL as at HL (CA4 phenotypic group 1; Figure 6), CA4-A strains exhibited more variability with three possible behaviors: i) a lower Exc_495:545_ ratio at HL than LL (group 2), ii) a delay in the initiation of the CA4 process after a shift from BL to GL (group 3) or iii) a reduced amplitude of variation of the ratio at LL and no variation at HL (group 4). The difference between groups 1 and 2 at HL could be due to the loss of one or two distal PEII discs from the phycobilisome rods of CA4-A strains, resulting in a lower PUB to PEB fluorescence excitation ratio, as previously observed in other *Synechococcus* strains grown at high white irradiance [38,39]. This hypothesis is consistent with the longer acclimation time needed to reach the maximal Exc_495:545_ ratio in HBL for CA4-A than for CA4-B strains (data not shown). 

CA4-A and CA4-B islands share the presence of two contiguous genes (*fciAB*) encoding putative transcriptional regulators and one gene coding for a characterized (MpeZ [20]; or a putative (MpeW) phycobilin lyase of the E/F clan [30], which is either on the same or the reverse strand as *fciAB* (Figure 1). MpeW is not orthologous to MpeZ since the best hit of both sequences is MpeY, a paralog of both proteins present in all PEII-containing *Synechococcus* strains (Figure 3). However, the combination of genomic and physiological data suggest that MpeW and MpeZ are mutually exclusive enzymes that could share the same function, i.e. to catalyze the attachment of a PEB chromophore at Cys-83 of the PEII alpha-chain (MpeA) and to isomerize it into PUB [20]. Yet, the fact that *mpeW* exhibited an opposite expression pattern in BL *vs.* GL with regard to *mpeZ* (see Figure 5 and [20] suggests that i) MpeW could be a mere PEB lyase although there are no obvious candidate gene in CA4-B strains coding for a PEB lyase-isomerase (equivalent to MpeZ in CA4-A strains) and/or ii) that the underlying regulatory machinery partially differs between the two types of CA4 processes. Transcriptional regulators of the AraC family are found in a wide range of microorganisms and regulate a variety of cellular pathways by activating or repressing transcription from cognate promoter [40]. Although most are encoded by a single gene (e.g. in the case of AraC itself; [27,41]), occurrence of a cluster of two *araC*-like genes, as is the case for *fciAB*, has also been reported in the case of rhamnose catabolism, which was shown to be regulated in a two-step activation cascade. In the presence of rhamnose, RhaR first induces *rhaSR* expression, causing an accumulation of RhaS that secondarily induces expression of the *rhaBAD* operon [42,43]. By analogy, it is likely that FciA and/or FciB regulate the transcription of *mpeZ* and *mpeW* in response to a signal induced by a change in the ambient light quality. The short *fciC* gene, which is found in CA4-A but not in CA4-B islands and that codes for a ribbon-helix-helix domain-containing protein previously found in bacterial and phage repressors [28], might also well play a role in the differential expression pattern of these two genes. Interestingly, *Synechococcus* sp. BIOS-E4-1 was found to possess a partial CA4-A island lacking the *fciAB* genes (Figure 1), providing us some additional insights into the putative regulatory role of these genes. Indeed, this strain is seemingly locked into the BL phenotype (Exc_495:545_^~^ 1.65), independently of the ambient light color (Figure 4) and its *mpeZ* is not differentially expressed between GL and BL (Figure 5). This suggests that in CA4-A strains in which *fci* genes are functional, the regulatory complex they encode might repress the expression of the *mpeZ* gene in GL. 

Several other *Synechococcus* strains exhibited no significant changes of their Exc_495:545_ ratio between BL and GL, indicating that their CA4 process is affected at either structural or regulatory levels. WH8016 constitutes another obvious example of the first type of alteration, since this strain lacks *mpeZ* and has a stop codon in *fciA*, resulting in a permanently low PUB phenotype (Figure 1). Four strains that possess a complete CA4-A or B island were also completely unable (WH8109, MVIR-18-1, WH8103) or only partially (RCC307) capable to chromatically acclimate (Figures 1 and 4; Table 1), suggesting that these strains could be natural mutants for this process. The case of RCC307 is particularly interesting in this context, since this strain exhibits small and reversible changes of the Exc_495:545_ ratio upon light color shifts, and the amplitude of variation is similar to that previously reported for a RS9916 mutant in which *mpeZ* was inactivated [20]. However, transcript accumulation data showed that this gene is significantly expressed in RCC307. Although it was only about 6-fold more expressed in BL than GL conditions, this was quite comparable to the difference observed in BIOS-U3-1, a typical CA4-A strain (Figure 5). Thus, it is possible that the expression of other, still unidentified, phycobilin lyase gene(s) involved in the chromophorylation of the two other cysteinyl binding sites that change during CA4 (i.e., Cys-139 α-PEI and Cys-140 α-PEII; [20]) could also be affected in RCC307. Another noteworthy example is MVIR-18-1, which despite having a complete CA4-A island, displayed a very low and invariable Exc_495:545_ ratio (Figure 5), comparable to that of the pigment type 3a strain WH7803, which has been shown to have a molar PUB:PEB ratio of 0:5 for PEI and 1:5 for PEII [44]. Interestingly, the PE gene region of MVIR-18-1 resembles that of other CA4-A strains, except that it lacks the *mpeU* gene (Figure S2), which codes for an uncharacterized putative phycobilin lyase of the E/F clan [30]. This suggests that MpeU could have a critical role for the PUB chromophorylation of phycoerythrins. Lastly, WH8103 and WH8109 that possess both a complete PE region (Figure S2) and a complete CA4-B island, also showed no differential expression of *mpeW* between GL and BL, probably explaining their inability to chromatically acclimate. Given their different phenotypes (Exc_495:545_ ratios of ^~^ 1.00±0.05 and 0.71±0.03, respectively; Figure 1), it is possible that these strains are affected at different levels of the CA4 regulatory network. 

### CA4-A and CA4-B island types have different evolutionary histories

CA4 appears to be widely distributed within the marine *Synechococcus* radiation. Indeed, CA4 strains are found in both subclusters 5.1 and 5.3, while none has been reported yet in the strictly coastal subcluster 5.2. As for genes of the phycobilisome region [7], phylogenetic analyses using genes of the CA4 island(s) are globally inconsistent with those made with classical marker genes, including the 16S rRNA [45], the 16S-23S rRNA internal transcribed sequence (ITS; [46], the cytochrome b_6_ subunit (PetB; [24]) or diverse combinations of concatenated core proteins [24,25,47]. This suggests that both CA4 island types have been laterally transferred between lineages at some point during the evolution of marine *Synechococcus*. This hypothesis is strengthened by the fact that CA4 regions exhibit a significantly lower GC % (*P*<0.01) than the whole genomes, i.e., 40.6 ± 2.5 % and 57.7 ± 3.4 % (n=15), respectively (Table S3). 

Acquisition of such CA4 islands by lateral transfer can potentially confer the ability to chromatically acclimate to strains that previously had a fixed phycobilisome pigmentation, but this most likely requires that the recipient strain possesses other genes found in the phycobilisome region of CA4 strains, as is the case for pigment type 3c strains (see Figure 4 in [7]). In contrast, strains exhibiting simpler pigment types likely miss too many phycobilisome genes to be able to chromatically acclimate after acquiring a CA4-B island. Strain WH8016 might represent an example of such a failed lateral transfer. Its phycobilisome region is clearly that of a pigment type 3a strain (Figure S2) and this is independently confirmed by phylogenetic analyses of various phycobilisome genes, such as *cpeY* or *mpeY* (Figure 3), which unambiguously group WH8016 with WH7803. The absence of *mpeZ* and the presence of a stop codon in *fciA* in WH8016 (Figure 1) may thus appear as signs of degeneration of the likely useless CA4 island, although we cannot exclude that the transfer was initially incomplete and/or imperfect. 

In order to gain more insights about how the different *Synechococcus* lineages have acquired the CA4 process by obtaining one or the other island type, we examined their genomic context. This environment is remarkably conserved around CA4-B islands, which are always located in the middle of the phycobilisome rod region and more precisely at the 3'-end of the PEII subregion (Figure S2). It is worth noting that CA4-B strains possess the longest phycobilisome region reported so far (about 30 Kbp) and we suggest it represents the ultimate degree of sophistication of this highly specialized genomic region. Indeed, the length of the phycobilisome region seems to have expanded during evolution as the complexity of *Synechococcus* phycobilisome rod composition and chromophorylation progressively increased [7]. Contrary to CA4-B islands, the genomic context around CA4-A islands varies widely from clade to clade, though it is fairly constant within clades (Figure 1). Interestingly, *psbA* or *hli* genes, which encode respectively the D1 protein of photosystem II and high light-inducible proteins, are frequently found immediately upstream or downstream of the latter island. In the case of RS9916, for instance, a complete *psbA* sequence is found upstream of the CA4-A island while a partial *psbA* sequence, 100% identical to the former, is found downstream. Both *psbA* and *hli* are highly conserved genes and known to be hotspots for intragenomic homologous recombination [48]. Both are also frequently found in cyanophages and are known to have a key role in phage-host interactions during infection [49-52]. Thus, this suggests that the CA4-A island might allow a more dynamic transmission of the CA4 process than its CA4-B counterpart, the occurrence of recombination hotspots in its close vicinity facilitating its genetic transfer by conjugation or via viral intermediates [48,49].

Altogether, results from this study can be integrated into a coherent evolutionary scenario in which an ancestral CA4 island would have initially occurred by duplication and divergence of a previously existing PEB lyase-isomerase gene, possibly the common ancestor of *mpeW, Y* and *Z*, followed by the acquisition of a *fciA/B-*like gene, coding for an AraC-like transcriptional regulator, which was also duplicated before divergence of the two gene copies. The distribution of the two island types among phylogenetic clades (CA4-A island in clades I, IV, IX and CRD1; CA4-B island in clades II, III and subcluster 5.3) as well as phylogenetic analyses of CA4 genes, which consistently split apart these two groups of strains (Figures 2 and S3), suggest that the CA4-B island appeared prior to the differentiation of subclusters 5.1 and 5.3, whereas the CA4-A island would have occurred within subcluster 5.1 but before the divergence of clades I, IV, IX and CRD1. Although the presence of the CA4-A island in RCC307 (subcluster 5.3) might seem at odd with this scenario, phylogenetic analyses of CA4 genes, which consistently place them very close to those of RS9916 (subcluster 5.1, clade IX; Figures 2-3 and S2), strongly suggest that this occurrence results from a fairly recent lateral transfer of a CA4-B island from a clade IX strain to RCC307 or one of its close ancestors. 

### A revised classification of Synechococcus pigment types

A further outcome from this work is that it allowed us to refine the classification of *Synechococcus* pigment types initially proposed by [7]) and which was based on the phycobiliprotein composition of phycobilisome rods: PC only in type 1; PC and PEI in type 2; PC, PEI and PEII in type 3. Additionally, the Exc_495:545_ ratio measured in LBL and LGL was used to delineate four subtypes (a through d) within pigment type 3 [7]. 

Based on the phenotypic characterization performed in the present study, we propose i) to set the limit of type 3a to <0.6 to fit the complete range of Exc_495:545_ values observed in MVIR-18-1 (Figure 4), ii) to set the range of Exc_495:545_ ratio for pigment type 3b (0.6 ≤ Exc_495:545_ <1.6) to take into account CA4 (and possibly pigment type 3c) strains altered in their PUB chromophorylation, and iii) to distinguish CA4 strains displaying a high (type 3d) or low (type 3e) amplitude of variation of the Exc_495:545_ ratio during a shift from LGL to LBL and reciprocally (Table 1). Additionally, we propose to complement this phenotype by genomic information by adding the suffix A or B to indicate the CA4 island type, when present, even for strains not capable of chromatic acclimation, likely due to various alterations that totally or partially block the CA4 process. For instance, MVIR-18-1 is now classified as 3aA and MINOS11 as 3dB. Note that RCC307 and WH8103, initially classified as 3b and 3c, are now referred to as 3eA and 3bB, respectively. This refined classification, allowing scientists to better describe the whole complexity and wide diversity of *Synechococcus* pigmentation, should constitute a useful tool to study and characterize the evolution, functioning and regulation of light-harvesting in marine cyanobacteria. 

## Materials and Methods

### Comparative genomics and sequence analysis

Genomic regions used in this study were obtained from public complete or draft genomes as well as preliminary unassembled contigs of 26 newly marine *Synechococcus* genomes sequenced by the Genoscope (Evry, France; Table S1). The latter strains, obtained from the Roscoff Culture Collection (http://www.sb-roscoff.fr/Phyto/RCC/index.php; [53], were first cloned using the pour plate technique with inclusion in the seawater-based solid medium PCRS11 [54] supplemented with 0.35% low melting point agarose (Invitrogen, Carlsbad, CA) to obtain single colonies. Strains were considered as clonal after three successive rounds of plating. DNA was then extracted from 2L of culture harvested when the level of contamination was minimal, as assessed by flow cytometry [55]. Cell pellets were resuspended by strong vortexing in TE (10 mM Tris, 1 mM EDTA, pH 8.0), lysed by incubation at 37°C during 1 h in presence of lysozyme (final concentration of 1 mg.mL^-1^), then treated 5 h at 55°C with proteinase K and SDS (final concentrations of 0.2 mg.mL^-1^ and 0.5 w/v, respectively). Nucleic acids were extracted consecutively with phenol: chloroform: isoamyl alcohol (25:24:1) and chloroform: isoamyl alcohol (24:1). Residual RNA was removed by RNase A treatment (final concentration of 100 µg.mL^-1^) for 30 min at 37°C. Nucleic acids were then submitted to another phenol:chloroform extraction, then precipitated with 0.6 vol of isopropanol and 0.65 M of NaCl at -20°C. Nucleic acids were recovered by centrifugation, washed consecutively with 100 % and 70 % ethanol (v/v), resuspended in TE prior purification and concentration using Amicon Ultra-4 (100,000 MWCO, Millipore, Molsheim, France). DNA was quantified using Qubit (Invitrogen, Carlsbad, USA) before sequencing using the Illumina technology starting from both pair-end (2 x 100bp) and mate-pair (2 x 100 bp, insert size: 4-10 kbp) libraries. Assembly into contigs was performed using the CLC AssemblyCell software (CLCBio, Prismet, Denmark).

CA4 gene regions extracted from these genomes were deposited in the GenBank nucleotide sequence database under the following accession numbers: KF177881-KF177905 (Table S1), after manual annotation using the Artemis software [56] and the Cyanorak v2.0 information system (http://www.sb-roscoff.fr/cyanorak). The presence of genes involved (or potentially involved) in chromatic acclimation was checked in marine *Synechococcus* genomes, by BLASTP searches [57] using BioEdit v7.0.9.0 [58] against all 43 available marine *Synechococcus* genomes. Presence of protein domains and motifs was checked using InterProScan v4.8 on the EMBL-EBI database, SMART 7 [59] and Phyre2 [26]. 

### Phylogenetic analyses

Sequences were aligned using MAFFT (G-INS-I option, v 6.953 [60]; with gap opening penalty and offset set at default values, and 1000 iterations. Poorly aligned regions and gaps were removed by trimAL (v1.4 ; [61]. Phylogenetic reconstructions were performed using 4 different methods: Neighbor Joining (NJ), Maximum Parsimony (MP), Maximum likelihood (ML) and Bayesian inference (BI). NJ and PARS analyses were performed using Phylip 3.69 as previously described [62]. ProtTest 3.2 was used for selection of best-fit models of amino acid replacement [63] according to the Akaike information criterion (AIC; [64] and Bayesian Information Criterion (BIC; [65]). ML reconstructions were performed using PhyML (v3.0 [66]; with the Le and Gascuel substitution model with an estimated Γ distribution parameter (LG+G model; [67], 1000 bootstrap replicates, four substitution-rate categories and a BIONJ starting tree. 

 Posterior probability values (PP) were generated using MrBayes (v3.1.2; [68], a mixed-model option, a swap frequency of 1, with four chains, a random starting tree and sampling every 100th generation. PP were generated from 1,000,000 generations for Unk10 and the first 100,000 generations were removed as burn-in. For phycobilin lyases and proteins containing an AraC-type domain, 1,500,000 generations were performed and the first 1,500 trees were discarded. Trees were visualized using FigTree v1.3.1 (http://tree.bio.ed.ac.uk/software/figtree/).

### Experimental growth conditions

Strains were grown at 22±1 °C in 50 mL polycarbonate culture flasks (Sarsted, Nümbrecht, Germany) in the seawater-based medium PCR-S11 [54], supplemented with 1 mM NaNO_3_. Seawater was reconstituted from Red Sea Salts^TM^ (Houston, Texas, USA) using distilled water. Colored filters (Lee, Andover, Hants, UK) were used to cover plastic boxes surrounding culture flasks in order to expose them either to blue (Mikkel BLue, #716, 448 nm peak transmission, 422-484 nm half-height width) or green light (Jas Green, #738, 511 nm peak transmission, 488-593 nm half-height width). Continuous light was provided by Daylight F58W/54-765-T8 fluorescence tubes (Sylvania, Gennevilliers, France). Experiments were performed under both LL (20±2 µmol photons m^-2^ s^-1^) and HL (75±5 µmol photons m^-2^ s^-1^), as measured with a quantameter QSL-2100 (Biospherical Instruments, San Diego, CA).

### Kinetics measurements, control of growth and optical properties

Marine *Synechococcus* strains were acclimated to the different experimental light conditions for at least one month, corresponding to at least 20 and 40 generations in LL and HL, respectively (see results). For kinetics studies, an exponentially growing batch culture (2.10^7^ to 2.10^8^ cells.mL^-1^) was diluted into fresh medium at a final concentration of ^~^10^6^ cells mL^-1^, then split into three subcultures. For each strain acclimated in BL or GL, one subculture was kept in the original condition (control), whereas the two experimental subcultures were shifted to the other light color condition, when the fluorescence excitation signal was sufficiently high to get useable Exc_495:545 nm_ values, i.e. in general immediately after dilution for LL cultures and 24 h after inoculation for HL ones. Measurements were then carried out every day in LL and every 12 h in HL. Fluorescence excitation spectra were recorded for an emission at 575 nm using a Perkin Elmer LS-50B spectrofluorimeter and used to calculate the fluorescence Exc_495:545_ ratio, a proxy to the molar PUB to PEB ratio. Moreover, for determining cell concentrations, a 500 µL aliquot was fixed with glutaraldehyde (0.25% final concentration, Grade II, Sigma Aldrich, Saint-Louis, MO, USA), incubated for 10 min in the dark and frozen at -80°C until flow cytometric analyses. Thawed samples were analyzed after dilution into sterile medium, using a FACS Canto (Becton Dickinson Biosciences, San Jose, CA, USA) in the presence of 0.95 µm standard fluorescent microspheres (Polysciences Warrington, PA, USA) as previously described [55]. Growth rates (µ in d^-1^) were computed as the slope of a Ln(N*t*) vs. time plot, where N*t* is the cell concentration at time *t*. The half acclimation time (T_50_ in d) was calculated using the regression wizard of SigmaPlot (v. 12, Systat Software, San Jose, CA) by fitting a sigmoidal non-linear regression to data with the following equation: 

f(x) = min + (*max-min*) / (1 + 10^logT50-x^ x a), where a is the slope of the curve. Minimum (min) and maximum (max) values were defined as average values of the Exc_495:545_ ratio of the GL and BL controls, respectively. 

Kinetics of acclimation over successive shifts was also monitored in *Synechococcus* strain BL107. During the whole experiment, the two biological replicates were maintained in exponential growth phase by diluting cultures every two days to a final concentration of ~8.10^6^ cells.mL^-1^. The dilution did not affect growth and chromatic acclimation rates. 

### Real time quantitative PCR

Real time PCR (qPCR) was used to monitor the expression level of *mpeZ* or *mpeW* genes in LGL and LBL acclimated cultures of 12 *Synechococcus* strains. Three independent exponentially growing cultures per strain were grown at a density of approximately 1-5 10^7^ cells mL^-1^. Cells were then harvested and RNA extracted as previously described [69]. Briefly, after addition of Pluronic F68 solution (0.01 % final concentration; Sigma Aldrich), 150 mL cultures were centrifuged at 9000 x *g* and 4°C for 7 min. Cell pellets were resuspended in 500 µL Trizol (Invitrogen, Carlsbad, CA, USA), immediately frozen in liquid nitrogen and stored at -80°C until RNA extraction. Samples were kept on ice during all steps. RNA extractions were performed using the miRNAeasy kit (Qiagen), following the manufacturer's instructions, in order to recover both large and small RNAs. Most residual DNA was removed by three successive DNase treatments on miRNeasy columns with the Qiagen RNase-free DNase (Qiagen). RNA samples were eluted in DEPC-treated water, quantified using a NanoDrop 1000 spectrophotometer (Thermo Scientific, Wilmington, DA), and kept at -80°C until analysis.

Primer Express^TM^ (v2.0; Applied Biosystems, Foster City, CA) was used to design gene-specific primers (Table S4) and optimization was performed by checking for each primer set its specificity and efficiency as assessed by standard curves generated using diluted cDNA with a maximum dynamic range, as previously described [70,71]. Primers were used at a final concentration of 300 mM, except for RS9915 *mpeW* and *rnpB* in all strains for which 600 mM was used. Reverse transcription was carried out using SuperScriptII reverse transcriptase (Gibco-BRL, Gaithersburg, MD, USA) on 100 ng RNA. qPCR analyses were performed using the 480 II (Roche, Boulogne-Billancourt, France) and the absolute SYBR Green ROX Mix (Abgene, Epsom, UK) using for each data point three biological and three technical replicates, corresponding to independent cultures and qPCR measurements, respectively. Reactions were incubated for 15 min at 95°C, followed by 45 cycles of 10 s at 95°C, 10 s at 60°C and 15 s at 72°C, terminated by a ramping from 65 to 97°C in order to perform a fusion curve. Quantification of the relative fold change in mRNA levels was made using the 2^-ΔΔCT^ method [72] using *rnpB* as a reference gene to normalize the relative transcript levels. 

## Supporting Information

Figure S1 **Multiple sequence alignments of putative transcriptional regulators FciA (**A**) and FciB (**B**), both containing an AraC-like α-helix-turn-α-helix (HTH, IPR018060) domain, and FciC (**C**) with a predicted ribbon-helix-helix (IPR010985 and IPR013321).** Shading represents identical amino acid in at least 70% of the sequences. Protein domains, framed by a black rectangle, were determined using InterProScan against *Synechococcus* sp. RS9916 sequences, used as reference. *Ab* *initio* modeling using Phyre2 indicated that 77% of FciA (263 of 342 residues) and 50% of FciB (158 of 316 residues) from *Synechococcus* sp. RS9916 could be modeled with >90% confidence to the AraC/XylS family. Furthermore, 78% of FciC (42 of 55 residues) were modeled based on the CopG-like family.
(PDF)

Figure S2 **Fraction of the phycobilisome rod gene region (including the whole PEII subregion) that varies between marine *Synechococcus* strains containing a CA4-A or B region, listed in black and blue, respectively.** Note that the gene organization of WH8016 phycobilisome region is identical to that of the pigment type 3a strain WH7803. See Figure 6 in [7] for examples of complete phycobilisome regions.
(PDF)

Figure S3 **Bayesian analysis of Unk10 (108 aa positions) from marine *Synechococcus*.** For each strain phylogenetic affiliation in mentioned into brackets and the pigment type is indicated by colored circles. The tree is rooted using the sequence from *Crocosphaera watsonii* sp. WH8501. Series of four numbers shown at nodes correspond to Bayesian posterior probabilities (PP, ranging between 0 and 1), bootstrap values for ML analyses, Neighbor-Joining and Parsimony methods, respectively. Bootstraps, represented as a percentage, were obtained through 1,000 repetitions and PP from 1,000,000 generations. Only values higher than 0.60 for PP and 60% for bootstrap values are shown on the phylogenetic tree. The scale bar represents 0.1 substitutions per nucleotide.
(PDF)

Figure S4 **Conserved motifs for phycobilin lyases members of the E/F clan of marine *Synechococcus*, as predicted by Protomata learner**(v 0.07, http://tools.genouest.org/
tools/protomata/learn/). E/Z-repeats motifs shared by these proteins are highlighted in blue. An additional motif of unknown function is indicated in pink. All sequences available for each protein were used for motif design. Numbers between brackets refer to the minimum and maximum number of residues between the different motifs.
(PDF)

Figure S5 **Effect of successive color shifts on the kinetics of chromatic acclimation in *Synechococcus* sp. BL107.** (A) Effects of the time of BL acclimation on the delay of CA4 initiation, as assessed by significant changes in the Exc_495:545_ ratio. For this experiment, GL acclimated cultures were first shifted to BL and once they had reached the Exc_495:545_ ratio in BL, they were shifted back to GL either immediately or after 1 or 3 days; (B) Two successive shifts from BL acclimated cultures. Cultures were shifted to GL and once they had reached minimal Exc_495:545_ values, cultures were shifted back to BL immediately, or after 1 or 3 days. Finally, cultures were again shifted to GL once they had reached the maximal Exc_495:545_ values.
(PDF)

Figure S6 **Effect of successive color shifts on the kinetics of chromatic acclimation in *Synechococcus* sp. BL107.** (A) Effects of the time of BL acclimation on the delay of CA4 initiation, as assessed by significant changes in the Exc_495:545_ ratio. For this experiment, GL acclimated cultures were first shifted to BL and once they had reached the Exc_495:545_ ratio in BL, they were shifted back to GL either immediately or after 1 or 3 days; (B) Two successive shifts from BL acclimated cultures. Cultures were shifted to GL and once they had reached minimal Exc_495:545_ values, cultures were shifted back to BL immediately, or after 1 or 3 days. Finally, cultures were again shifted to GL once they had reached the maximal Exc_495:545_ values.
(PDF)

Table S1 **Characteristics of marine *Synechococcus* strains and accession numbers of sequences used in this study.**
(XLSX)

Table S2 **Kinetics of acclimation of twelve marine *Synechococcus* strains after shifting cultures to the other light color.** Growth rate (µ) were assessed from flow cytometric counts while T_50_ was calculated from Exc_495:545_ spectrofluorimetric measurements. Values indicated for individual strains are averages of two biological replicates (± mean deviation) calculated from two biological replicates. Averages (± standard deviation) were also calculated for the different sets of strains gathered by CA4 type or physiological behavior group (see text).
(XLSX)

Table S3 **Comparison of the G + C content in CA4 regions vs. whole genomes.**
(XLSX)

Table S4 **List of primers used for real time PCR reactions.**
(XLSX)
