# Supplementary material for: A Gene Island with Two Possible Configurations Is Involved in Chromatic Acclimation in Marine Synechococcus
Source: PLoS One. 2013 Dec 31;8(12):e84459. doi: 10.1371/journal.pone.0084459 (PMC3877281; doi:10.1371/journal.pone.0084459)
Supplement: Figure S1 — Multiple sequence alignments of putative transcriptional regulators FciA (A) and FciB (B), both containing an AraC-like α-helix-turn-α-helix (HTH, IPR018060) domain, and FciC (C) with a predicted ribbon-helix-helix (IPR010985 and IPR013321). Shading represents identical amino acid in at least 70% of the sequences. Protein domains, framed by a black rectangle, were determined using InterProScan against Synechococcus sp. RS9916 sequences, used as reference. Ab initio modeling using Phyre2 indicated that 77% of FciA (263 of 342 residues) and 50% of FciB (158 of 316 residues) from Synechococcus sp. RS9916 could be modeled with >90% confidence to the AraC/XylS family. Furthermore, 78% of FciC (42 of 55 residues) were modeled based on the CopG-like family. (PDF) [file pone.0084459.s001.pdf]

SUPPLEMENTARY INFORMATION

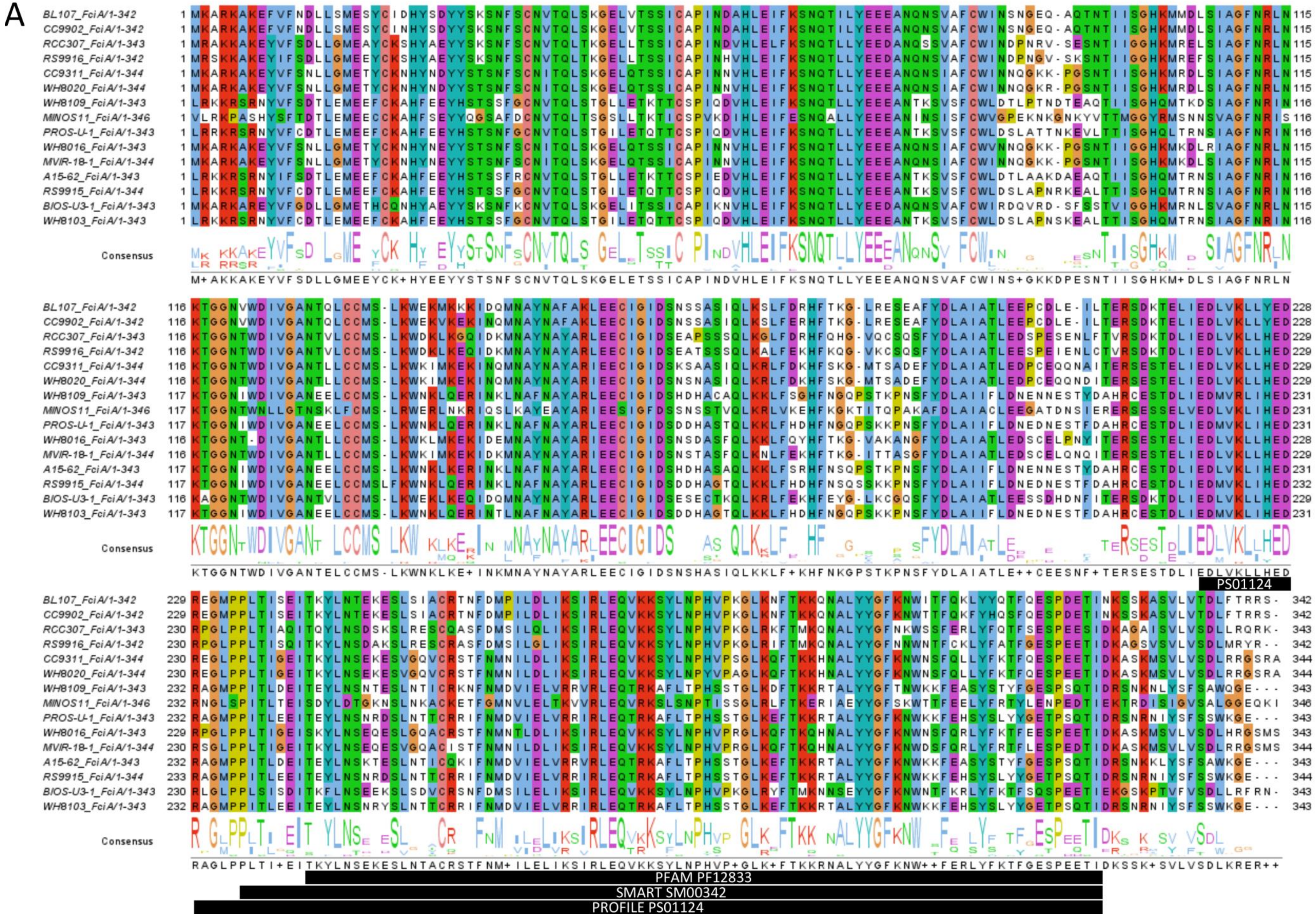

B

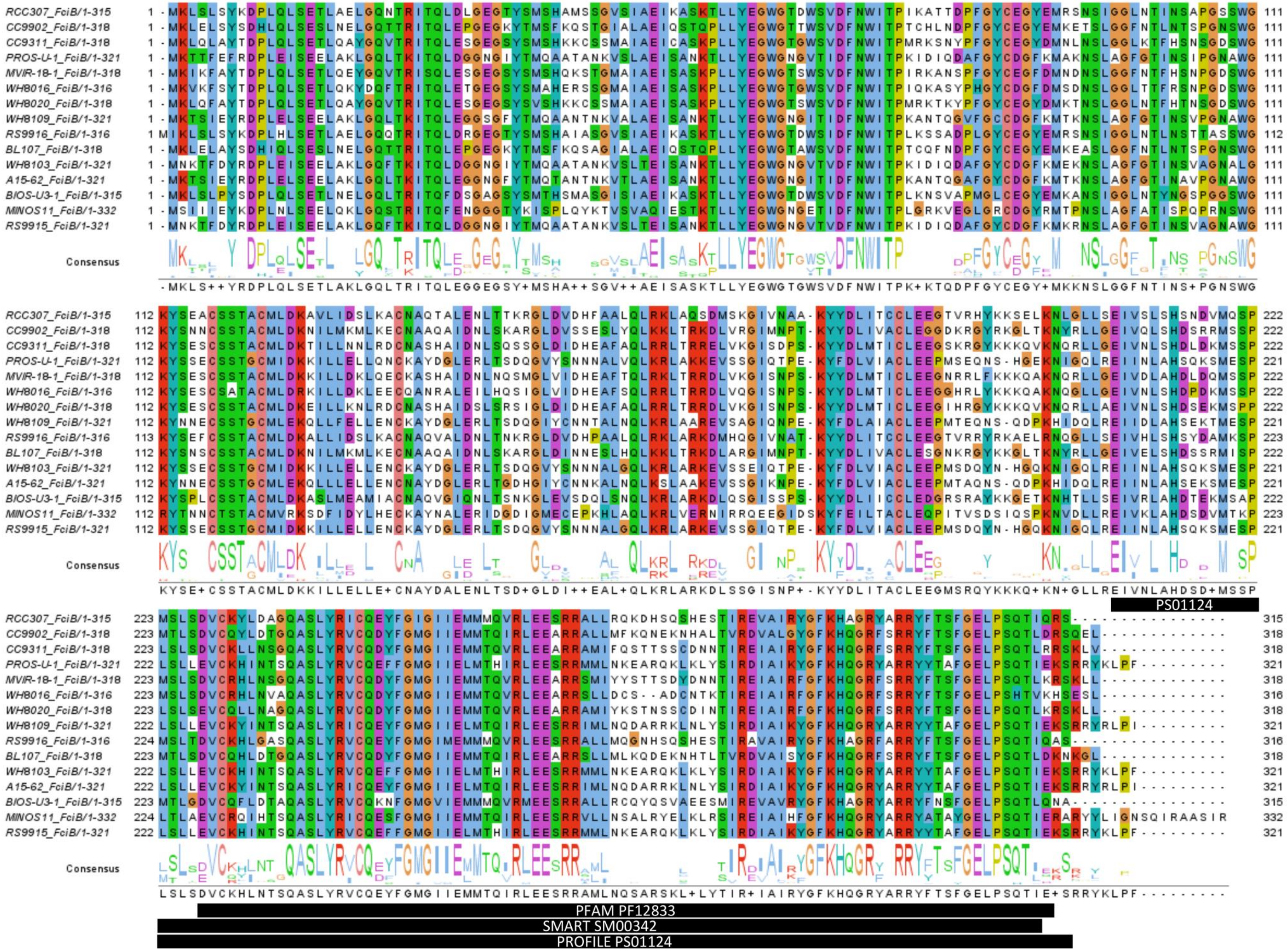
RCC307_FciB/1-315
CC9902_FciB/1-318
CC9311_FciB/1-318
PROS-U-1_FciB/1-321
MVIR-18-1_FciB/1-318
WH8016_FciB/1-316
WH8020_FciB/1-318
WH8109_FciB/1-321
RS9916_FciB/1-316
BL107_FciB/1-318
WH8103_FciB/1-321
A15-62_FciB/1-321
BIOS-U3-1_FciB/1-315
MINOS11_FciB/1-332
RS9915_FciB/1-321
Consensus
PS01124
PFAM PF12833
SMART SM00342
PROFILE PS01124

C

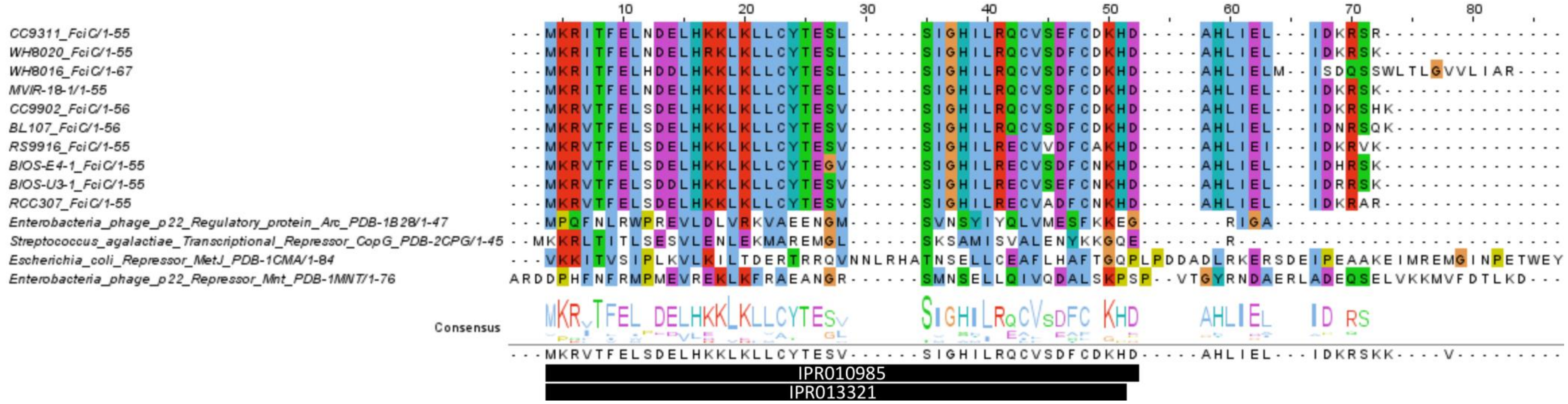

Figure S1. Multiple sequence alignments of putative transcriptional regulators FciA (A) and FciB (B), both containing an AraC-like α-helix-turn-α-helix (HTH, IPR018060) domain, and FciC (C) with a predicted ribbon-helix-helix (IPR010985 and IPR013321). Shading represents identical amino acid in at least 70% of the sequences. Protein domains, framed by a black rectangle, were determined using InterProScan against *Synechococcus* sp. RS9916 sequences, used as reference. *Ab initio* modeling using Phyre2 indicated that 77% of FciA (263 of 342 residues) and 50% of FciB (158 of 316 residues) from *Synechococcus* sp. RS9916 could be modeled with >90% confidence to the AraC/XylS family. Furthermore, 78% of FciC (42 of 55 residues) were modeled based on the CopG-like family (Gomis-Rüth et al., 1998).

Gomis-Rüth, F.X., Solà, M., Acebo, P., Párraga, A., Guasch, A., Eritja, R. et al. (1998) The structure of plasmid-encoded transcriptional repressor CopG unliganded and bound to its operator. *EMBO J* **17**(24): 7404-7415.
