# Supplementary material for: A Gene Island with Two Possible Configurations Is Involved in Chromatic Acclimation in Marine Synechococcus
Source: PLoS One. 2013 Dec 31;8(12):e84459. doi: 10.1371/journal.pone.0084459 (PMC3877281; doi:10.1371/journal.pone.0084459)
Supplement: Figure S2 — Fraction of the phycobilisome rod gene region (including the whole PEII subregion) that varies between marine Synechococcus strains containing a CA4-A or B region, listed in black and blue, respectively. Note that the gene organization of WH8016 phycobilisome region is identical to that of the pigment type 3a strain WH7803. See Figure 6 in [7] for examples of complete phycobilisome regions. (PDF) [file pone.0084459.s002.pdf]

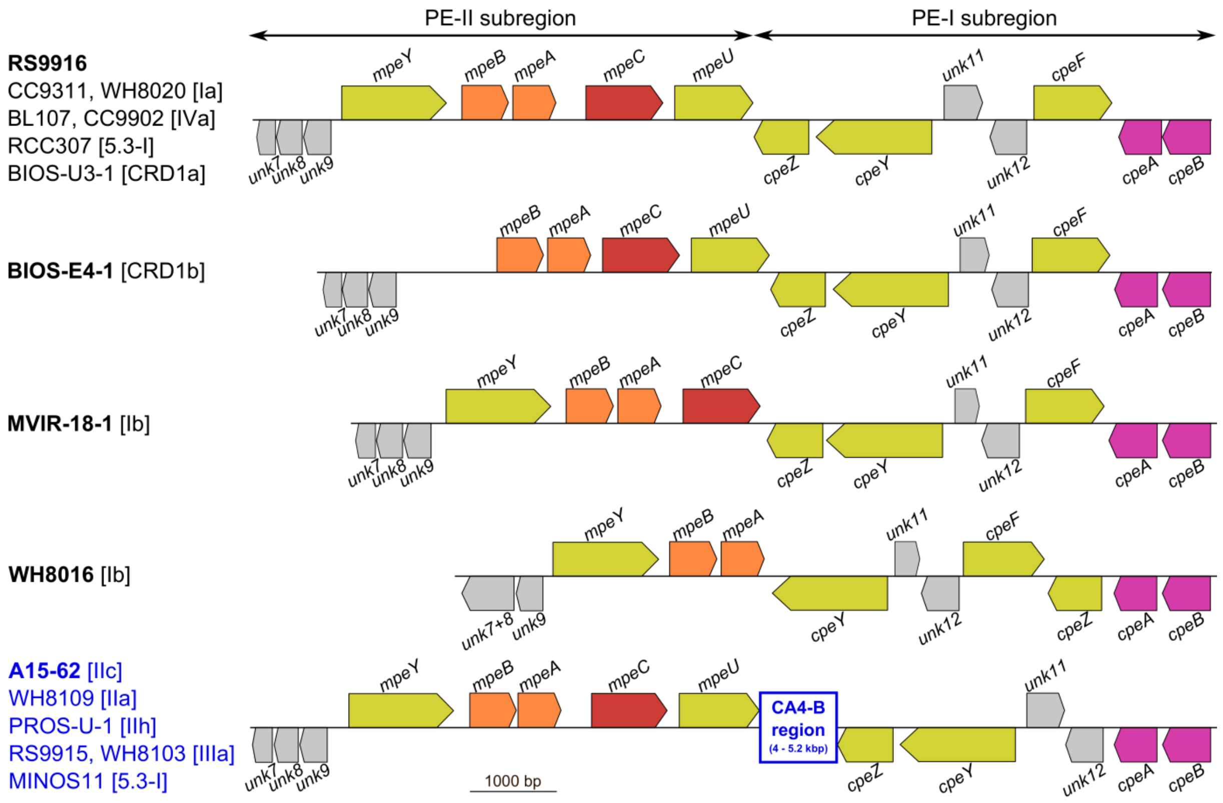

Figure S2. Fraction of the PBS rod gene region (including the whole PEII subregion) that varies between marine *Synechococcus* strains containing a CA4-A or B region, listed in black and blue, respectively. Note that the gene organization of WH8016 PBS region is identical to that of the pigment type 3a strain WH7803. See Fig. 6 in Six et al. (2007).
