# Supplementary material for: A Gene Island with Two Possible Configurations Is Involved in Chromatic Acclimation in Marine Synechococcus
Source: PLoS One. 2013 Dec 31;8(12):e84459. doi: 10.1371/journal.pone.0084459 (PMC3877281; doi:10.1371/journal.pone.0084459)
Supplement: Figure S3 — Bayesian analysis of Unk10 (108 aa positions) from marine Synechococcus. For each strain phylogenetic affiliation in mentioned into brackets and the pigment type is indicated by colored circles. The tree is rooted using the sequence from Crocosphaera watsonii sp. WH8501. Series of four numbers shown at nodes correspond to Bayesian posterior probabilities (PP, ranging between 0 and 1), bootstrap values for ML analyses, Neighbor-Joining and Parsimony methods, respectively. Bootstraps, represented as a percentage, were obtained through 1,000 repetitions and PP from 1,000,000 generations. Only values higher than 0.60 for PP and 60% for bootstrap values are shown on the phylogenetic tree. The scale bar represents 0.1 substitutions per nucleotide. (PDF) [file pone.0084459.s003.pdf]

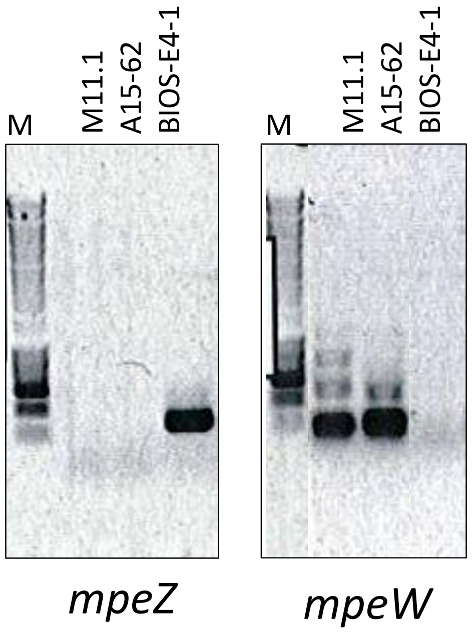

Figure S3. Electrophoresis gels showing the presence of *mpeW* in *Synechococcus* sp. M11.1. PCR amplification were performed using a GeneAmp PCR system 9700 (Applied Biosystems, Carlsbad, California) with an initial denaturation step of 5 min at 94 °C, followed by 35 cycles (30 s at 94 °C, 30 s at 50 °C and 1 min at 72 °C) and a final extension step of 10 min at 72 °C and using the following primers: mpeZ_2F, TGGCHGARMGWTTTGATGT; mpeZ_310R, GAYACNGAGGCYGTNATTA; mpeW_175F, KCYACYGGGGAYAGAGA and mpeW_960R, GAACRCCATARTCRGAKCCATG. DNA from strains A15-62 and BIOS-E4-1 were used as positive (*mpeW*-containing strain) and negative (*mpeZ*-containing strain) controls, respectively. M, DNA molecular weight marker (SmartLadder, Eurogentec).
