# Supplementary material for: A Gene Island with Two Possible Configurations Is Involved in Chromatic Acclimation in Marine Synechococcus
Source: PLoS One. 2013 Dec 31;8(12):e84459. doi: 10.1371/journal.pone.0084459 (PMC3877281; doi:10.1371/journal.pone.0084459)
Supplement: Figure S4 — Conserved motifs for phycobilin lyases members of the E/F clan of marine Synechococcus, as predicted by Protomata learner(v 0.07, http://tools.genouest.org/ tools/protomata/learn/). E/Z-repeats motifs shared by these proteins are highlighted in blue. An additional motif of unknown function is indicated in pink. All sequences available for each protein were used for motif design. Numbers between brackets refer to the minimum and maximum number of residues between the different motifs. (PDF) [file pone.0084459.s004.pdf]

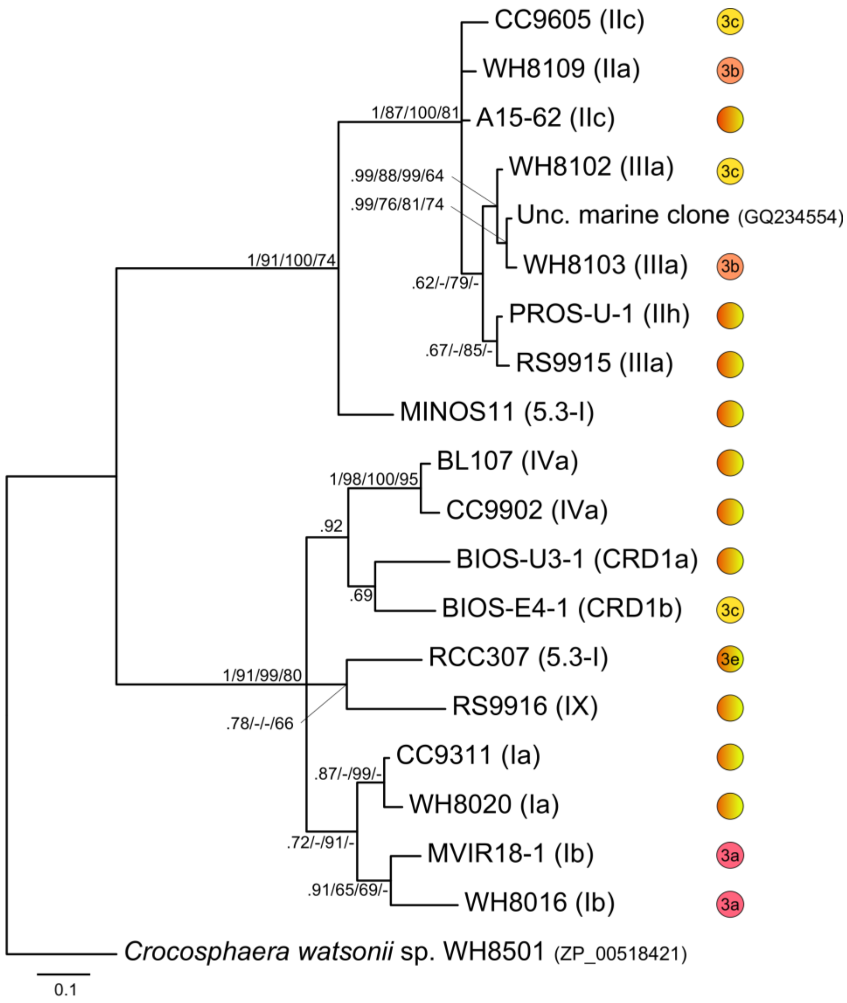

Figure S4. Bayesian analysis of Unk10 (108 aa positions) from marine *Synechococcus.* For each strain phylogenetic affiliation in mentioned into brackets and the pigment type is indicated by colored circles. The tree is rooted using the sequence from *Crocosphaera watsonii* sp. WH8501. Series of four numbers shown at nodes correspond to Bayesian posterior probabilities (PP, ranging between 0 and 1), bootstrap values for ML analyses, Neighbor-Joining and Parsimony methods, respectively. Bootstraps, represented as a percentage, were obtained through 1,000 repetitions and PP from 1,000,000 generations. Only values higher than 0.60 for PP and 60% for bootstrap values are shown on the phylogenetic tree. The scale bar represents 0.1 substitutions per nucleotide.
