# Supplementary material for: A Gene Island with Two Possible Configurations Is Involved in Chromatic Acclimation in Marine Synechococcus
Source: PLoS One. 2013 Dec 31;8(12):e84459. doi: 10.1371/journal.pone.0084459 (PMC3877281; doi:10.1371/journal.pone.0084459)
Supplement: Figure S6 — Effect of successive color shifts on the kinetics of chromatic acclimation in Synechococcus sp. BL107. (A) Effects of the time of BL acclimation on the delay of CA4 initiation, as assessed by significant changes in the Exc495:545 ratio. For this experiment, GL acclimated cultures were first shifted to BL and once they had reached the Exc495:545 ratio in BL, they were shifted back to GL either immediately or after 1 or 3 days; (B) Two successive shifts from BL acclimated cultures. Cultures were shifted to GL and once they had reached minimal Exc495:545 values, cultures were shifted back to BL immediately, or after 1 or 3 days. Finally, cultures were again shifted to GL once they had reached the maximal Exc495:545 values. (PDF) [file pone.0084459.s006.pdf]

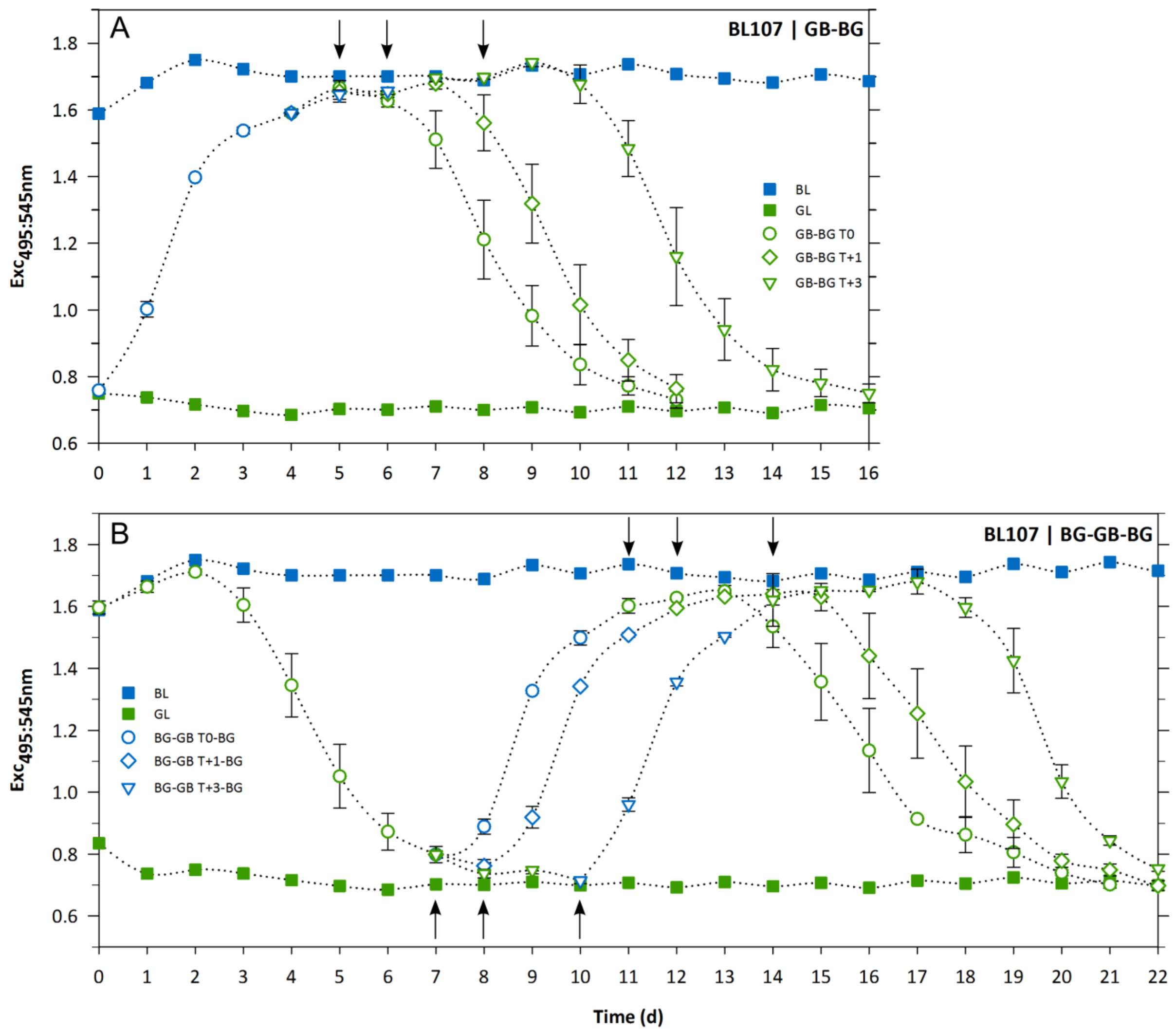

<u>Figure S6</u>. Effect of successive color shifts on the kinetics of chromatic acclimation in *Synechococcus* sp. BL107. (A) Effects of the time of BL acclimation on the delay of CA4 initiation, as assessed by significant changes in the $Exc_{495:545}$ ratio. For this experiment, GL acclimated cultures were first shifted to BL and once they had reached the $Exc_{495:545}$ ratio in BL, they were shifted back to GL either immediately or after 1 or 3 days; (B) Two successive shifts from BL acclimated cultures. Cultures were shifted to GL and once they had reached minimal $Exc_{495:545}$ values, cultures were shifted back to BL immediately, or after 1 or 3 days. Finally, cultures were again shifted to GL once they had reached the maximal $Exc_{495:545}$ values.
